# Indole-Based Compounds as Potential Drug Candidates for SARS-CoV-2

**DOI:** 10.3390/molecules28186603

**Published:** 2023-09-13

**Authors:** Adel S. Girgis, Siva S. Panda, Benson M. Kariuki, Mohamed S. Bekheit, Reham F. Barghash, Dalia R. Aboshouk

**Affiliations:** 1 Department of Pesticide Chemistry, National Research Centre, Dokki, Giza 12622, Egypt; m_bekheit@yahoo.com (M.S.B.); reham_fawzy@yahoo.com (R.F.B.); daliaraslan205@yahoo.com (D.R.A.); 2 Department of Chemistry and Biochemistry, Augusta University, Augusta, GA 30912, USA; 3 Department of Biochemistry and Molecular Biology, Augusta University, Augusta, GA 30912, USA; 4 School of Chemistry, Cardiff University, Main Building, Park Place, Cardiff CF10 3AT, UK; kariukib@cardiff.ac.uk (B.M.K.)

**Keywords:** indole, COVID-19, SARS-CoV-2, alkaloids, natural, synthetic, in silico

## Abstract

The COVID-19 pandemic has posed a significant threat to society in recent times, endangering human health, life, and economic well-being. The disease quickly spreads due to the highly infectious SARS-CoV-2 virus, which has undergone numerous mutations. Despite intense research efforts by the scientific community since its emergence in 2019, no effective therapeutics have been discovered yet. While some repurposed drugs have been used to control the global outbreak and save lives, none have proven universally effective, particularly for severely infected patients. Although the spread of the disease is generally under control, anti-SARS-CoV-2 agents are still needed to combat current and future infections. This study reviews some of the most promising repurposed drugs containing indolyl heterocycle, which is an essential scaffold of many alkaloids with diverse bio-properties in various biological fields. The study also discusses natural and synthetic indole-containing compounds with anti-SARS-CoV-2 properties and computer-aided drug design (in silico studies) for optimizing anti-SARS-CoV-2 hits/leads.

## 1. Introduction

**Figure 1:**
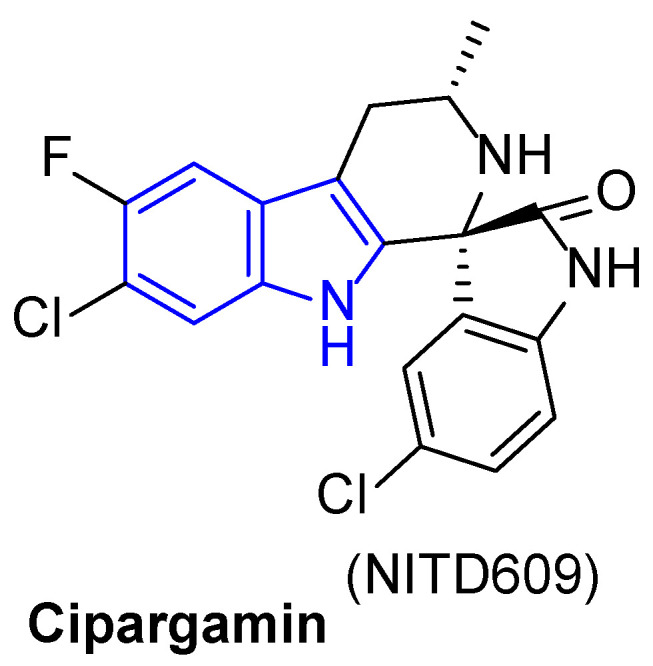
Cipargamin (NITD609) is in pre-clinical studies as a potential antimalarial drug [16].

**Figure 2:**
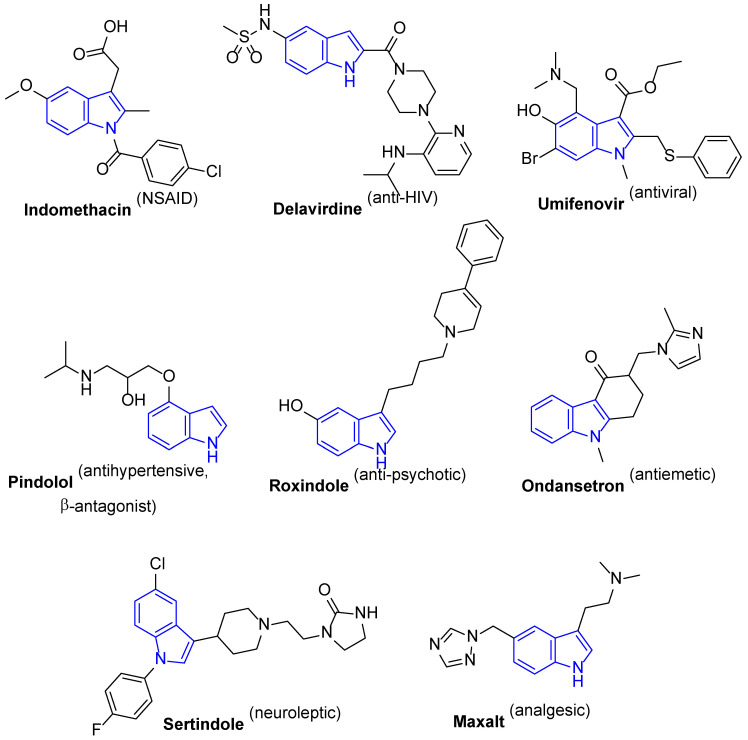
Indole-containing drugs against different diseases [29,30,31,32,33,34,35,36,37,38].

**Figure 3:**
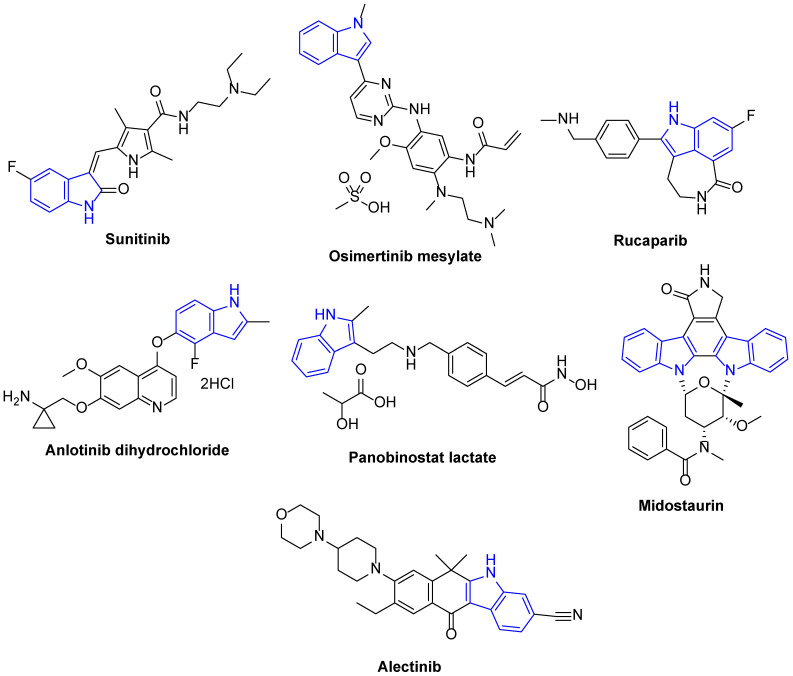
Indole-containing anticancer agents [40,41,42,43,44,45,46,47,48,49,50].

The indole moiety is one of the most privileged scaffolds in the alkaloid category. Indole-containing compounds are widely distributed in plants, animals, and microorganisms and represent important pharmacophores that can bind with receptors controlling bio-properties [1]. Diverse biological properties have been exhibited by natural and synthetic indole-containing analogs, including anti-microorganism activities such as antibacterial [2,3,4,5], antifungal [6,7,8], antiviral [9,10,11,12,13,14], and mycobacterial [15] action. Cipargamin (Figure 1), which has an indolyl scaffold, has been identified as a potent protein synthesis inhibitor in *Plasmodium falciparum* and has subsequently progressed to pre-clinical trials as a potential antimalarial drug [16]. Other potential antimalarial candidates with the indolyl scaffold have also been reported [17,18,19,20]. Anti-diabetic [21,22] and anti-inflammatory [23,24,25,26,27] properties have also been observed for indole derivatives. Figure 2 presents some of the indole-containing drugs approved for the treatment of a range of conditions [1,2,28,29,30,31,32,33,34,35,36,37,38]. Indole-based anticancer drugs and potent agents [28,39,40,41,42,43,44,45,46,47,48,49,50] are illustrated in Figure 3 and many indolyl analogs identified as antitumor-active candidates have also been reported [51,52,53,54,55,56,57].

The coronavirus disease 2019 (COVID-19) has proved to be one of the most serious crises facing human health in recorded history. The disease is caused by the fast-spreading infectious virus, SARS-CoV-2 (severe acute respiratory syndrome coronavirus-2), transmitted between humans and threatening human life worldwide. Until 2019, the virus strain had not been reported as invasive among humans [58]. The first infection was initially linked to a fish and wild animal market in Wuhan City, China, at the end of 2019 before the disease dramatically spread, within a few weeks, to almost all countries of the world, affecting millions of people [59]. The World Health Organization (WHO) declared a global severe emergency and pandemic in March 2020 [60]. To date (5 July 2023), WHO statistics have officially counted/confirmed 767.7 million infected patients with 6.949 million deaths worldwide [61].

The symptoms of COVID-19 are similar to those observed for many other conditions and seasonal diseases (flu is an example) and include cough, runny nose, mild fever, and headache. Breathing difficulties, chest pain, and hypertension occur in severe infections that require hospitalization in intensive care and oxygen supply [62,63]. The pandemic placed many countries under unprecedented economic pressure due to the curtailment of normal social activities that affected a significant amount of the global population. The scientific community, including research institutes, universities, and pharmaceutical companies, devoted considerable resources to studying the cell biology of SARS-CoV-2, identifying diagnostic agents, and optimizing effective therapeutics [64].

SARS-CoV-2 is a zoonotic single-strand RNA (ssRNA(+)) virus covered by glycoprotein spikes and belongs to the Coronaviridae family. It is mainly found in bats, but, for unknown reasons, was transmitted to the human species, leading to the global pandemic. The viral RNA genetic material can directly act as viral messenger RNA, producing the viral protein inside the host cell [65,66,67,68]. Numerous waves of viral mutations were detected, potentially affecting transmissibility and severity in humans. Delta (B.1.617) and Delta plus (B.1.617.2) are deadlier and more infectious than the other mutations (for example, Alpha, B.1.1.7; Beta, B.1.351; or Gamma, P.1) [62]. Omicron is more extensively mutated and was detected in November 2021 with the viral wave spreading worldwide. Although it has a higher risk of infection/reinfection, it has milder symptoms and lower fatality in vaccinated people. This may be a factor in the pandemic transitioning to an epidemic [69].

Vaccination is one of the most important means for controlling the development of a pandemic and saving human lives. The neutralizing antibodies from vaccination can protect against viral infection or at least result in milder/weaker symptoms upon infection. In the case of COVID-19, BNT162b2 (Pfizer “USA”/BioNTech “Germany”), mRNA-1273 (Moderna, MA, USA), and AZD1222 (University of Oxford “UK” and AstraZeneca “British-Swedish”) have been the most prominent vaccines discovered and used worldwide [60,69]. Herd immunity is the aim of large-scale vaccination, and it may limit the extent of infection and terminate a global pandemic. In the case of COVID-19, evidence for the acquired immunity in recovered patients was limited, raising questions about the herd immunity hypothesis. Consequently, the acquired immunity due to vaccination antibodies is not certain against infection/re-infection [59,70].

**Figure 4:**
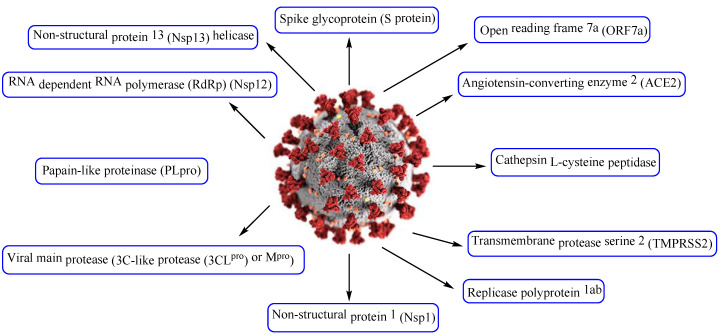
Proteins involved in the SARS-CoV-2 infection, which make them potential targets for optimizing therapeutics [71].

Biochemical studies have identified some proteins involved in SARS-CoV-2 infections (Figure 4 summarizes some) [71], and thus, are potential targets for controlling the infection and optimizing potential therapeutics.

The emerging healthcare crisis due to the global outbreak caused by the pathogenic SARS-CoV-2 virus united the scientific community and the pharmaceutical industry in a race against time in the search and optimization of any medicinal entity/device/therapeutic capable of controlling the spread of COVID-19 and bringing back normality [72]. De-novo drug development usually follows several successive steps. The first is the discovery of potent and safe agents from among many candidates. Preclinical studies support the biochemical mode of action and applicability of the agents as potentially therapeutic. Clinical trials are essential for assessing success and identifying side effects. The post-marketing safety monitoring step is necessary to continue the new therapeutics as one of the available medications. Drug development is, therefore, time-consuming and costly, requiring about 10–15 years and millions of dollars to progress from the pre-identification of the potent/lead compound through to the medical store. Only about 10% of the potential agents are successful [73,74,75].

**Figure 5:**
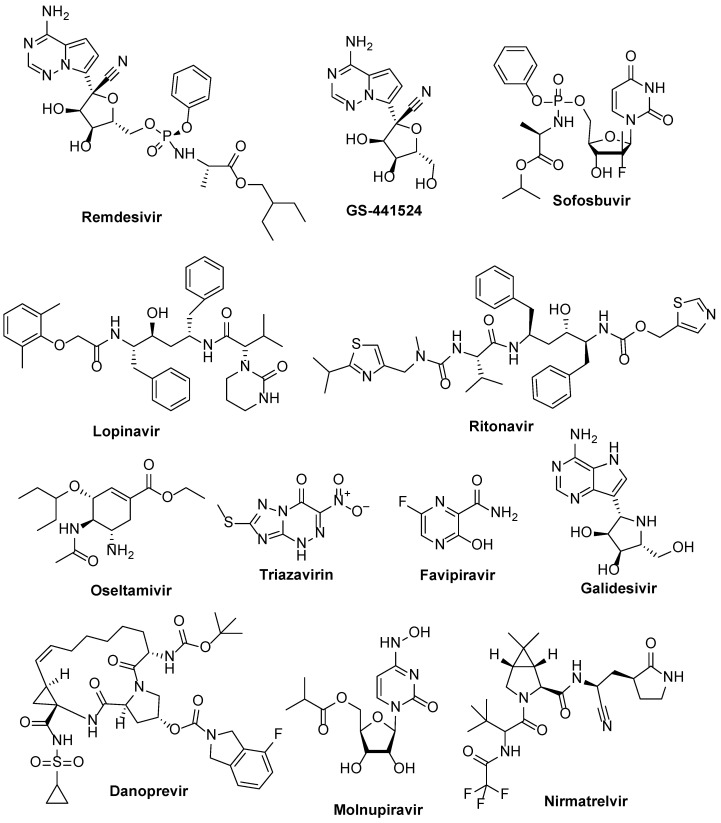
Repurposed antiviral agents with anti-SARS-CoV-2 properties [63,81,82,83,84,85].

**Figure 6:**
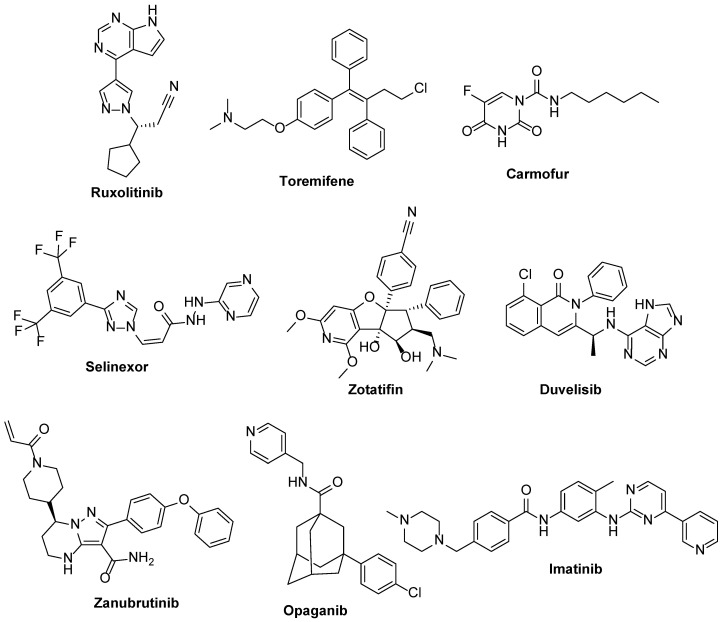
Repurposed anticancer agents with anti-SARS-CoV-2 properties [63,81,82,83,84,85].

**Figure 7:**
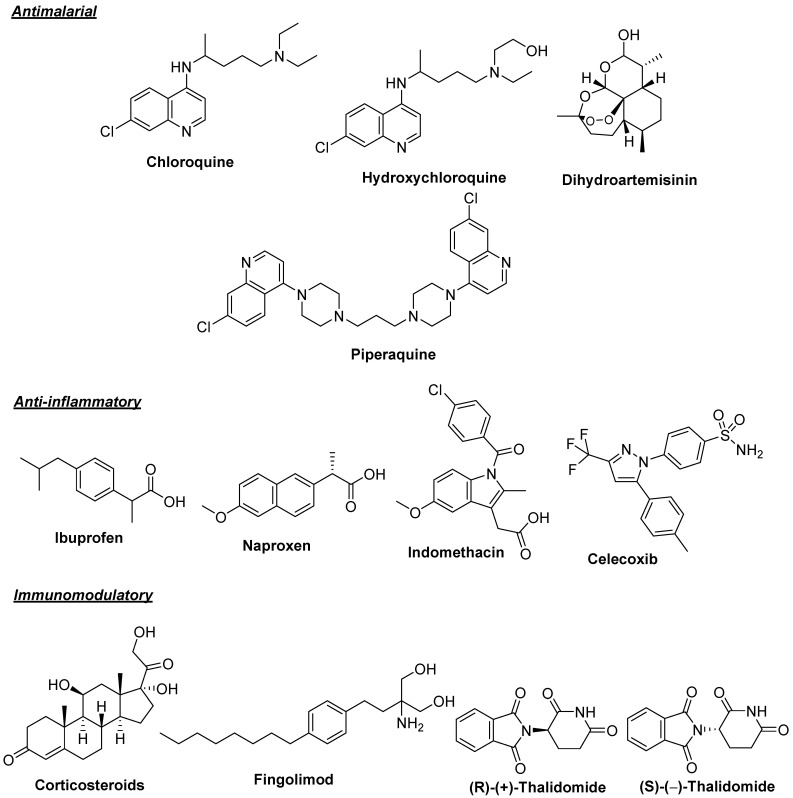
Repurposed antimalarial, anti-inflammatory, and immunomodulatory drugs with anti-SARS-CoV-2 properties [63,81,82,83,84,85].

In silico studies utilizing various techniques/software can assist in assigning anti-SARS-CoV-2 agents. This may shorten the time needed for identifying potential entities to be submitted for in vitro/in vivo testing followed by the clinical trial(s) phases. In other words, in silico studies are a shortcut to attaining the final targeted therapeutics, saving the time and money required [76,77]. Additionally, drug repurposing/re-profiling, a strategy that considers the use of approved or investigated drugs outside the original therapeutic application, can shorten the period needed due to the well-established safety profile and understood side effects/drawbacks. This approach has many advantages over developing entirely novel therapeutics, including cost reduction and lower risk [78,79,80]. Several antiviral (Remdesivir, GS-441524, Sofosbuvir, Lopinavir, Ritonavir, Oseltamivir, Triazavirin, Favipiravir, Galidesivir, Danoprevir, Molnupiravir, Nirmatrelvir), anticancer (Ruxolitinib, Toremifene, Carmofur, Selinexor, Zotatifin, Duvelisib, Zanubrutinib, Opaganib, Imatinib), antimalarial (Chloroquine, Hydroxychloroquine, Dihydroartemisinin, Piperaquine), anti-inflammatory (Ibuprofen, Naproxen, Indomethacin, Celecoxib), and immunomodulatory (Corticosteroids, Fingolimod, (R)-(+)-Thalidomide, (S)-(–)-Thalidomide) active agents/drugs have been repurposed for anti-SARS-CoV-2 application (Figure 5, Figure 6 and Figure 7) [63,81,82,83,84,85].

Remdesivir (Figure 5) is a broad-spectrum antiviral agent. It was the first therapeutic granted approval under emergency-use authorization by the Food and Drug Administration (FDA) [9,59,73]. Gilead Sciences originally developed it as an anti-Ebola virus agent. It was approved as a COVID-19 therapeutic with RNA-dependent RNA polymerase (RdRp) inhibitory properties due to its ability to be metabolized in the infected/host cell analogs to a nucleoside triphosphate. It can terminate viral replication through RdRp action via integration in the RNA viral chain [9,86]. Molnupiravir (Lagevrio) (Figure 5) was awarded FDA approval in December 2021 [87,88]. It also exerts its anti-SARS-CoV-2 activity via RdRp inhibition [9]. Paxlovid, a combination of Nirmatrelvir and Ritonavir (Figure 5), was awarded FDA approval in December 2021 [89]. Ritonavir/Pf-07321332 acts against SARS-CoV-2 through main protease (M^pro^) inhibition [83].

Our current discussion builds upon our previous work, which aimed to explore materials with anti-SARS-CoV-2 properties that could potentially aid in identifying agents against COVID-19 [9,66,90,91,92,93]. Specifically, we examine indole-containing compounds, whether they are naturally occurring or artificially created, that may possess anti-SARS-CoV-2 properties (Supplementary Materials Table S1).

## 2. Repurposed Indole-Containing Drugs

### 2.1. Umifenovir (Arbidol)

**Figure 8:**
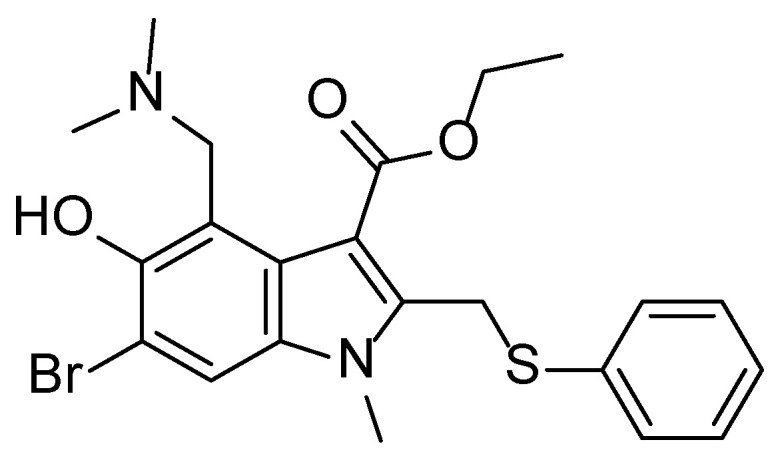
Umifenovir/Arbidol, repurposed drug for COVID-19.

Umifenovir (Arbidol) (Figure 8) is a broad-spectrum antiviral drug with inhibitory properties against both RNA and DNA viruses such as Zika, influenza, hepatitis (HBV, HCV), ebola, West Nile, and herpes viruses [94,95]. It is one of the drugs that has been repurposed against COVID-19 and has IC_50_ = 4.11 μM against SARS-CoV-2 [96]. It acts through the inhibition of the lipid envelope thereby limiting contact, and hence, the fusion of the viral cell (membrane fusion inhibitor) with the host/human cell (targeting S-protein/ACE2 “angiotensin-converting enzyme 2”) [97,98,99,100]. Computational studies including molecular docking (PDB ID: 6LZG) [101] and molecular dynamics [102,103] support the mode of action. The antioxidant properties of Arbidol have also been attributed to its ability to react with free radicals. This may indicate that the anti-SARS-CoV-2 bio-properties of Arbidol arise from several biochemical pathways [94]. Clinical studies have confirmed the suitability of Arbidol as a monotherapy or in combination with other therapeutics for COVID-19 patients [104,105,106,107,108,109,110,111,112,113,114]. Some countries (e.g., Russia, China, and Iran [95]) have awarded licenses to Arbidol for the prevention or treatment of COVID-19 [115].

**Scheme 1:**
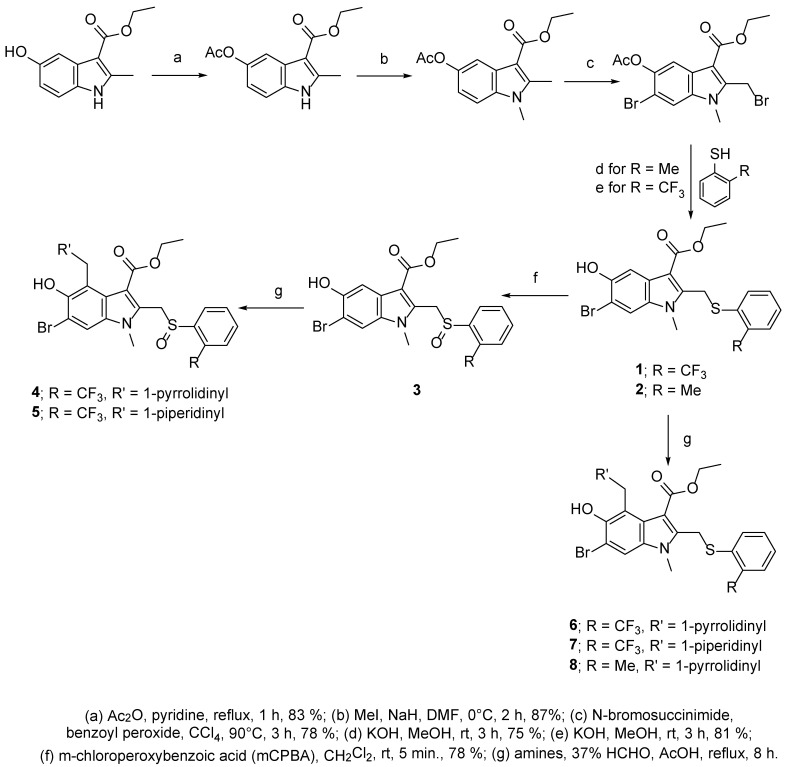
Synthesis of Arbidol analogs **1–8** of potential binding of the spike glycoprotein (S-protein, ACE2).

**Figure 9:**
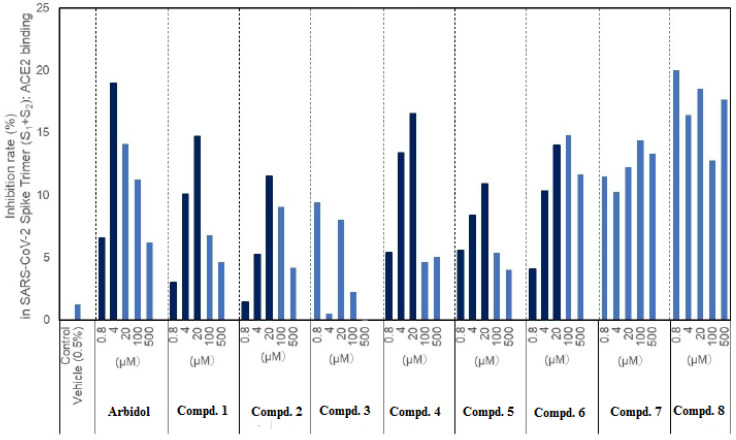
Inhibitory properties of Arbidol analogs **1**–**8**.

A series of Arbidol analogs **1**–**8** have been synthesized starting from 5-hydroxy-2-methylindole-3-carboxylate (Scheme 1). Potential binding of the compounds with the spike glycoprotein (S-protein, ACE2 binding) was determined (Figure 9) revealing no inhibition rates greater than 20%. Some inhibitory properties at low concentrations were higher than others but this can be explained in terms of the low solubility of the compounds in aqueous medium [96].

**Figure 10:**
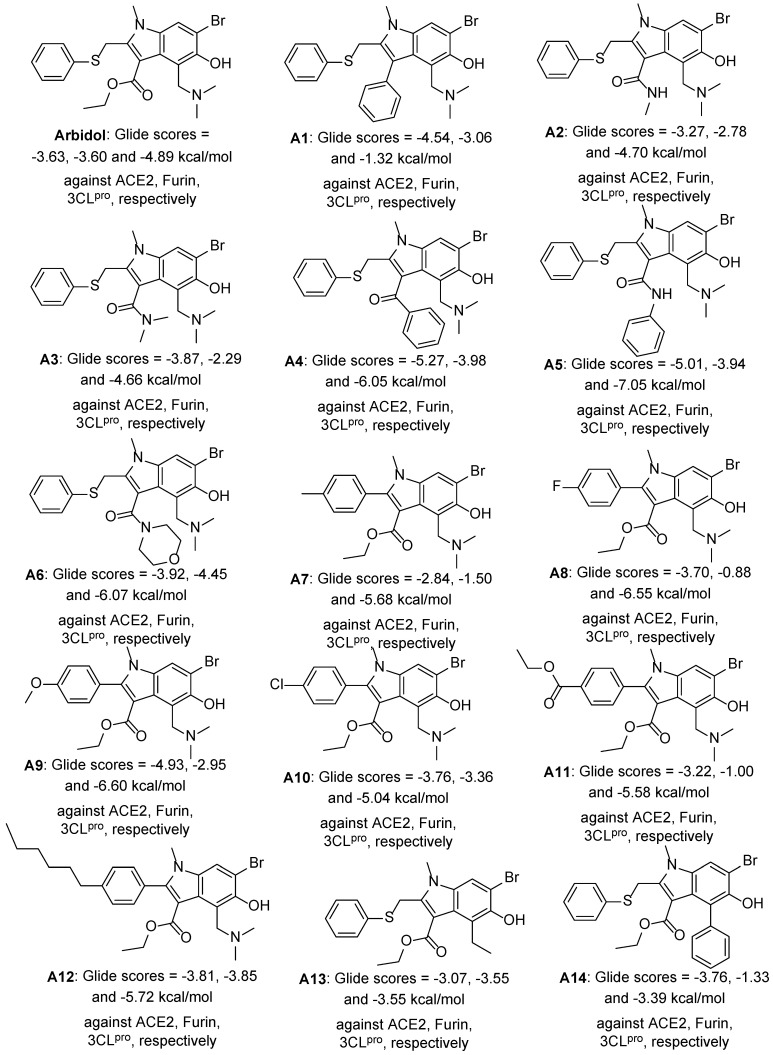

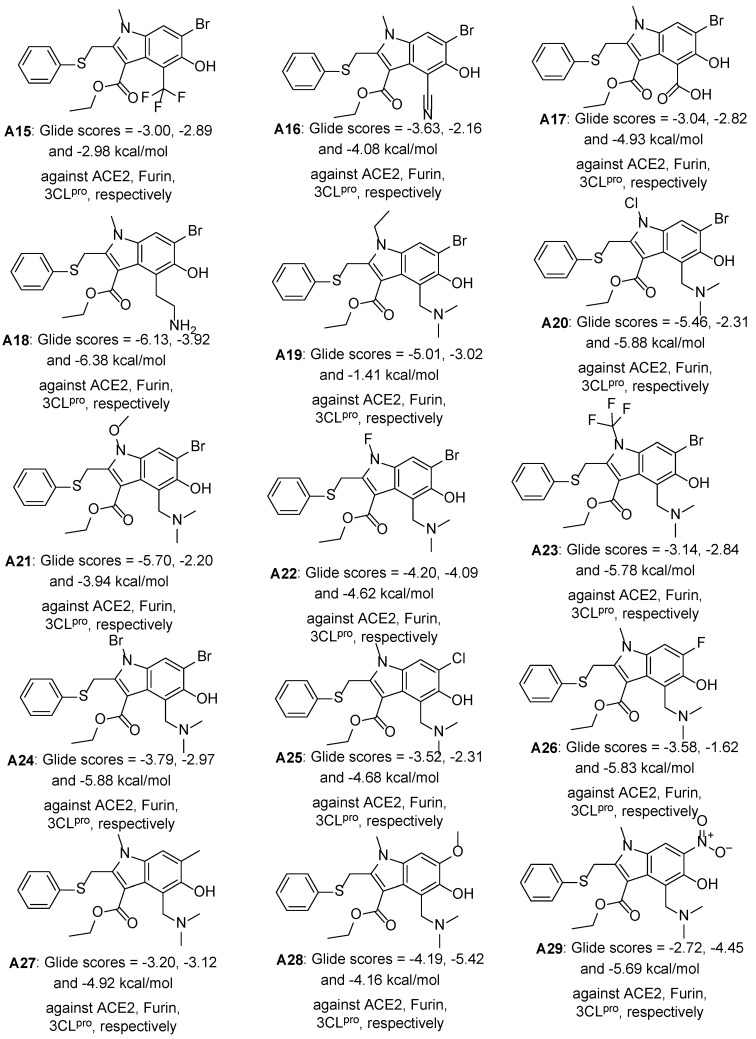

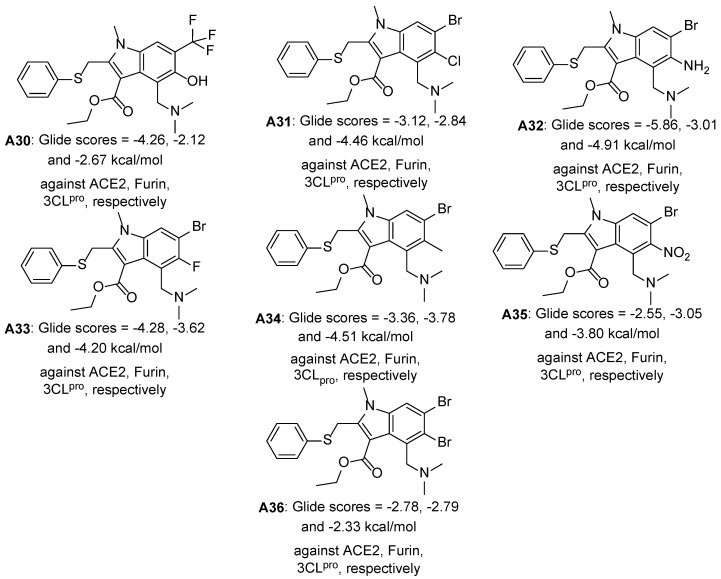
Glide scores predicted for Arbidol analogs against ACE2, Furin, and 3CL^pro^.

Arbidol analogs (**A1**–**A36**) have also been designed, through in silico studies using Schrodinger software, as inhibitors of ACE2, which is the key receptor that facilitates the entrance of the SARS-CoV-2 virus into the host cell (PDB ID: 6LZG) in addition to the proteases such as furin (PDB ID: 5MIM), TMPRSS2 (transmembrane protease serine 2), TMPS2 human, and 3CL^pro^ (3 chymotrypsin-like protease, PDB ID: 6LU7), which are essential for the viral replication. This approach may enable the optimization of multi-targeted inhibitor agents with potential efficacy against COVID-19, but the lack of experimental bio-properties data limits progress [116] (Figure 10).

### 2.2. Indomethacin

Indomethacin (Figure 7) is a non-steroidal anti-inflammatory (NSAID) and analgesic drug used worldwide [29,117,118]. It works through the non-selective inhibition of cyclooxygenase (COX), which is the key enzyme to produce prostaglandin from arachidonic acid. Prostaglandin is responsible for inflammation and pain [119]. Inflammation is a natural response of the human body due to harmful effects. It is associated with many diseases, including microorganism (bacterial/viral) infections, cancers, and asthma [90].

**Figure 11:**
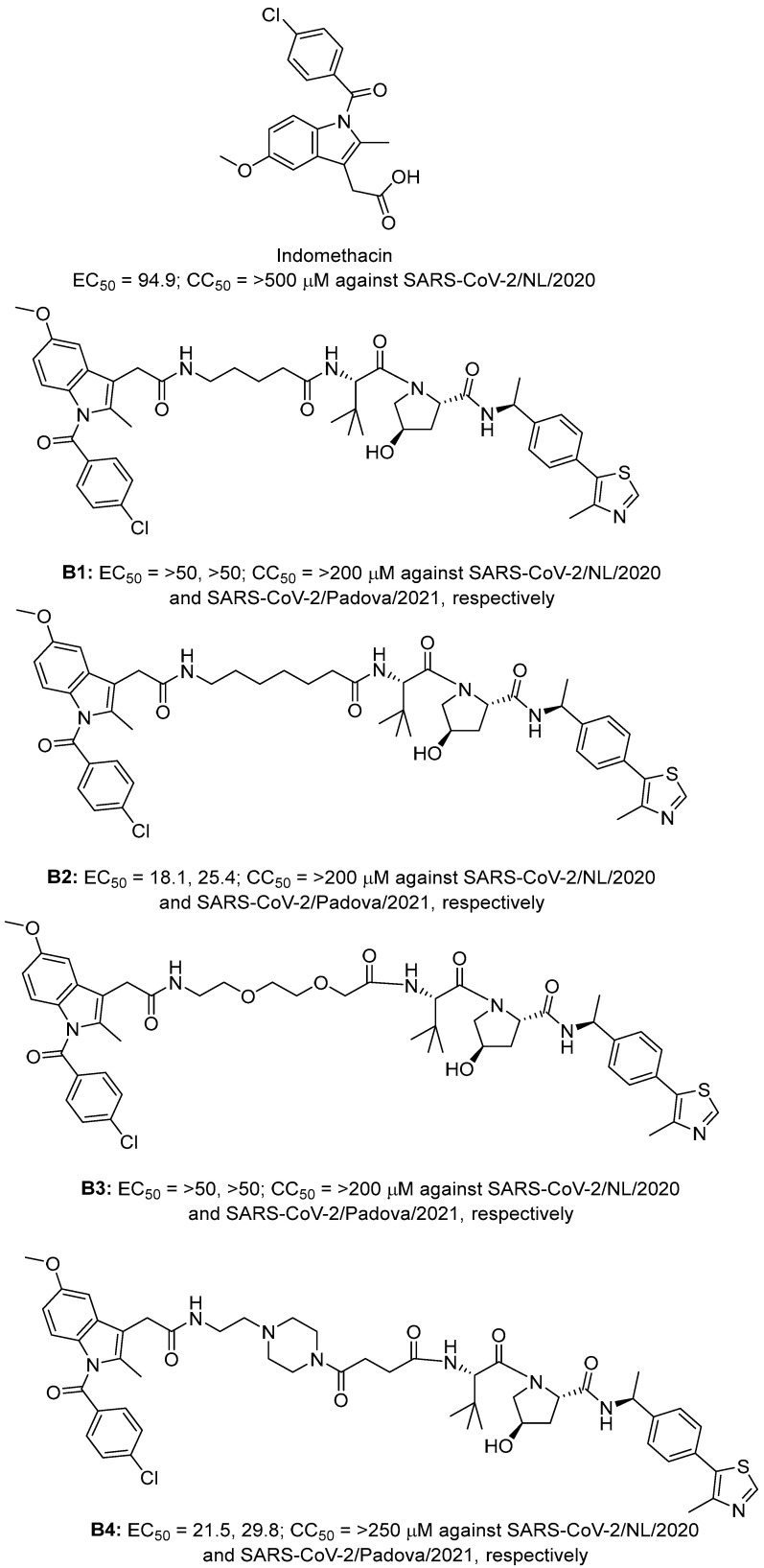
Indomethacin-Remdesivir conjugates (**B1**–**B4**) against SARS-CoV-2/NL/2020 and SARS-CoV-2/Padova/2021 strains [121].

Although indomethacin does not inhibit the replication of SARS-CoV-2 (infected Vero E6 bio-assay), studies have reported its potential as a co-treatment for COVID-19 patients due to its potent efficacy against symptoms associated with the disease [120,121,122,123,124]. Some Indomethacin-Remdesivir conjugates-based proteolysis-targeting chimeras (PROTAC) (**B1**–**B4**) have been reported with enhanced properties against SARS-CoV-2/NL/2020 and SARS-CoV-2/Padova/2021 strains relative to the parent indomethacin (EC_50_ = 94.9; CC_50_ > 500 µM against SARS-CoV-2/NL/2020) [121] (Figure 11). Conjugation between these drugs, or similar agents, can be a useful approach for optimizing promising hits/leads against SARS-CoV-2.

### 2.3. Lufotrelvir (PF-07304814)

**Figure 12:**
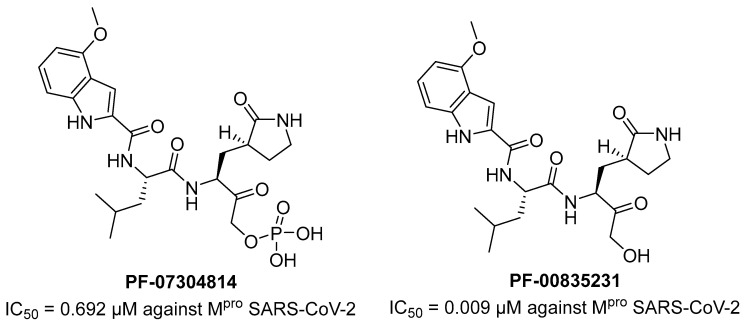
Lufotrelvir (PF-07304814) and its effective agent PF-00835231, M^pro^-SARS-CoV-2 inhibitor [125].

Lufotrelvir (PF-07304814) (Figure 12) is a SARS-CoV-2 main protease (M^pro^) inhibitor developed by Pfizer for intravenous application. The phosphate group is cleaved in vivo liberating PF-00835231, the effective agent against various viral strains [125].

### 2.4. Obatoclax (GX15-070)

**Figure 13:**
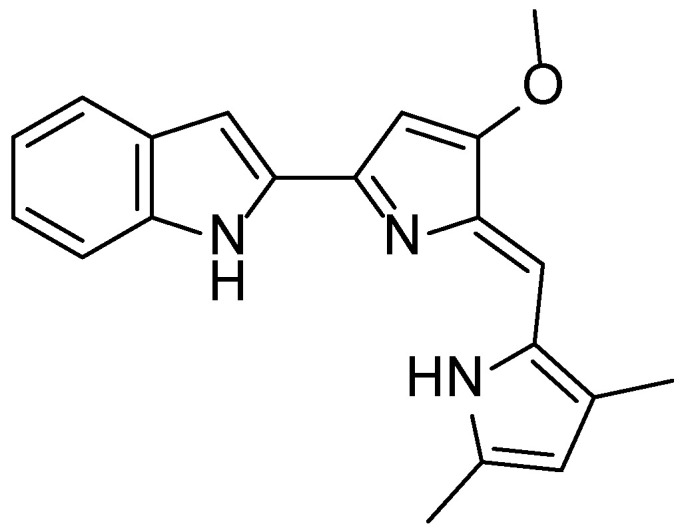
Obatoclax (GX15-070); EC_50_: 23.2 µM, repurposed drug for COVID-19 [126].

Obatoclax (GX15-070) (Figure 13) is an antitumor agent (leukemia, lymphoma, and lung) via BCL-2 protein inhibition inducing mitochondrial apoptosis and has been subjected to Phase II clinical trials. It was repurposed for COVID-19 due to its promising properties against ACE2, thereby blocking cellular entry by the infectious virus [126].

## 3. Natural Indole-Containing Compounds

### 3.1. Melatonin

**Figure 14:**
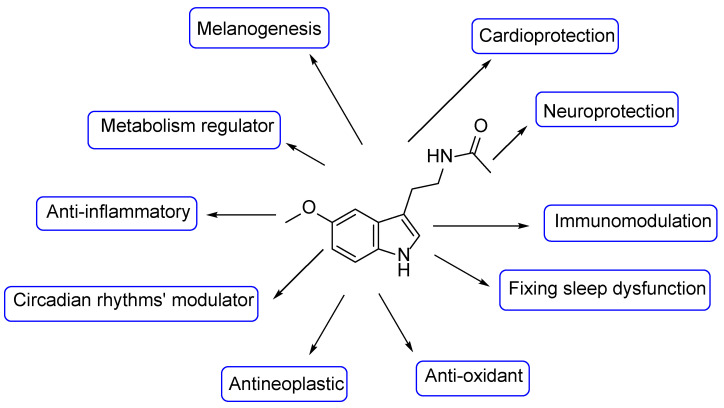
Main functions/properties of melatonin [127,128,129,130,131].

Melatonin is a natural hormone primarily biosynthesized from tryptophan by the pinealocytes of the pineal gland in the brain in the dark (hormone of darkness) and transferred by blood to the body organs from the cerebrospinal fluid. It exerts several biological properties [127,128,129,130,131] (Figure 14). The correlation between the COVID-19 fatalities in the elderly and the decrease in melatonin secretion rate drew attention to a possible application of the hormone for treatment [132]. The ability of melatonin as an antioxidant and anti-inflammation also suggested a potential role as an anti-SARS-CoV-2 [133]. Due to its safety profile and diverse bio-properties, numerous reports have considered the role of melatonin in preventing and treating COVID-19 [134,135,136,137,138,139]. Clinical studies/observations have supported its ability to reduce the severity of the disease, shorten the hospitalization period, or lead to complete recovery upon administration, either as a mono-therapeutic [137,138,139] or with other therapeutics, for COVID-19 infected patients [134,135,136].

### 3.2. Neoechinulin A, Echinulin, and Eurocristatine

**Figure 15:**
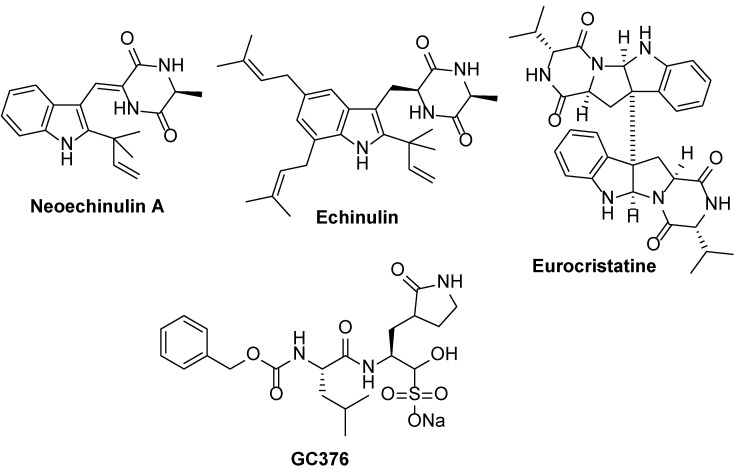
Natural neoechinulin A, echinulin, and eurocristatine from *Aspergillus fumigatus* MR2012 and GC376 (potent M^pro^-SARS-CoV-2 inhibitor) [140,141].

The natural compounds neoechinulin A, echinulin, and eurocristatine (Figure 15) can be obtained from organisms such as *Aspergillus fumigatus* MR2012. Neoechinulin A and echinulin have M^pro^-SARS-CoV-2 inhibitory properties (IC_50_ = 0.47, 3.90 μM, respectively) [140]. For comparison, the value is (IC_50_ = 0.36 μM) for GC376, a potent M^pro^-SARS-CoV-2 inhibitor [141].

**Scheme 2:**
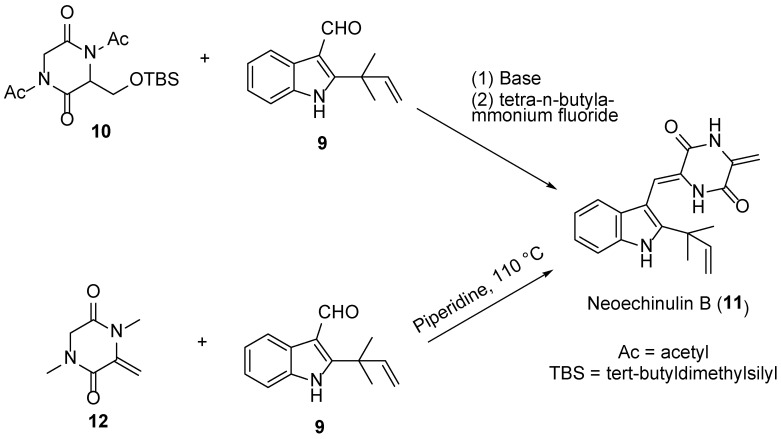
Synthesis of Neoechinulin B (**11**) of anti-SARS-CoV-2 properties [142].

Neoechinulin B **11** can be extracted from *Eurotium rubrum* Hiji025. It has been synthesized in a two-step reaction involving the 2-indole aldehyde **9** and the appropriate 2,5-piperazinedione **10** in basic conditions followed by tetra-*n*-butylammonium fluoride. Alternatively, **11** could be obtained from the aldehyde **9** and 2,5-piperazinedione **12** in the presence of piperidine at 110 °C (Scheme 2). Neoechinulin B **11** has anti-SARS-CoV-2 properties (Vero E6, assay, IC_50_ = 32.9, CC_50_ > 70 μM) [142].

## 4. Synthetic Indole-Containing Compounds

### 4.1. Isatins

Erdmann and Laurent first isolated isatin (1H-indole-2,3-dione) as an oxidation product of indigo using nitric and chromic acids. Isatin is found in humans as a metabolic derivative of the adrenaline hormone and a component of secretion from the parotid gland of Bufo frogs. Various isatin derivatives also naturally occur in plants, such as methoxy phenylpentyl isatins (the melosatin alkaloids) isolated from *Melochia tomentosa*, a Caribbean tumorigenic plant. Isatin and its derivatives are an important group of heterocyclic compounds that can serve as precursors for drug synthesis. Since its discovery, a significant amount of research has been conducted on the synthesis and biological and industrial applications of isatin.

**Scheme 3:**
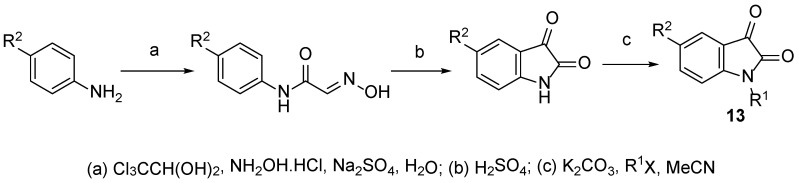
Synthesis of isatin derivatives **13** of potential 3CL^pro^ inhibitory properties [143].

**Figure 16:**
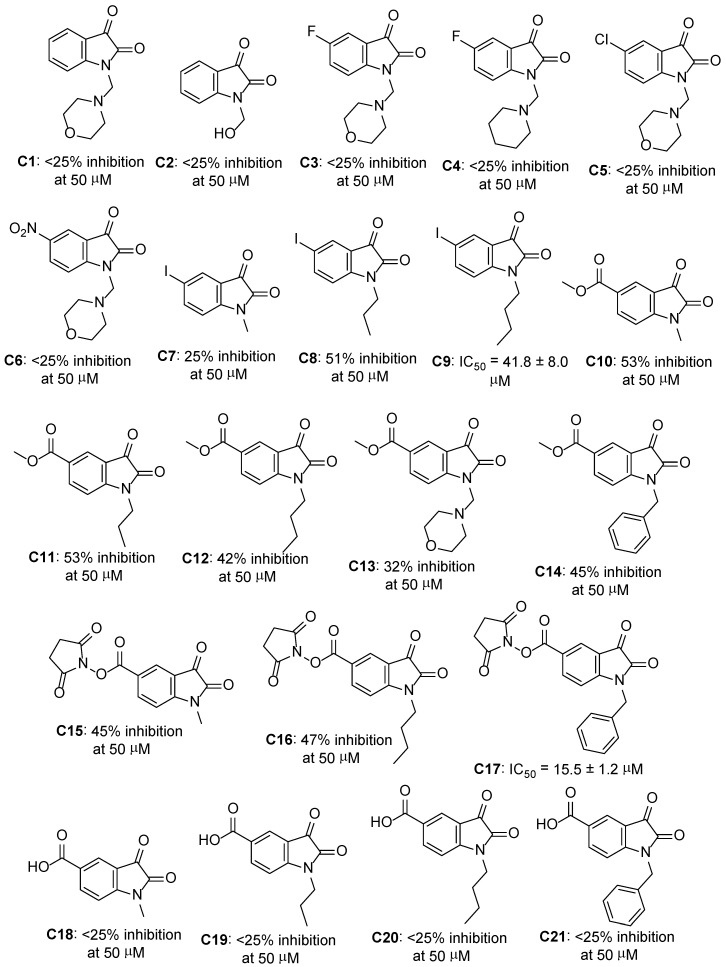

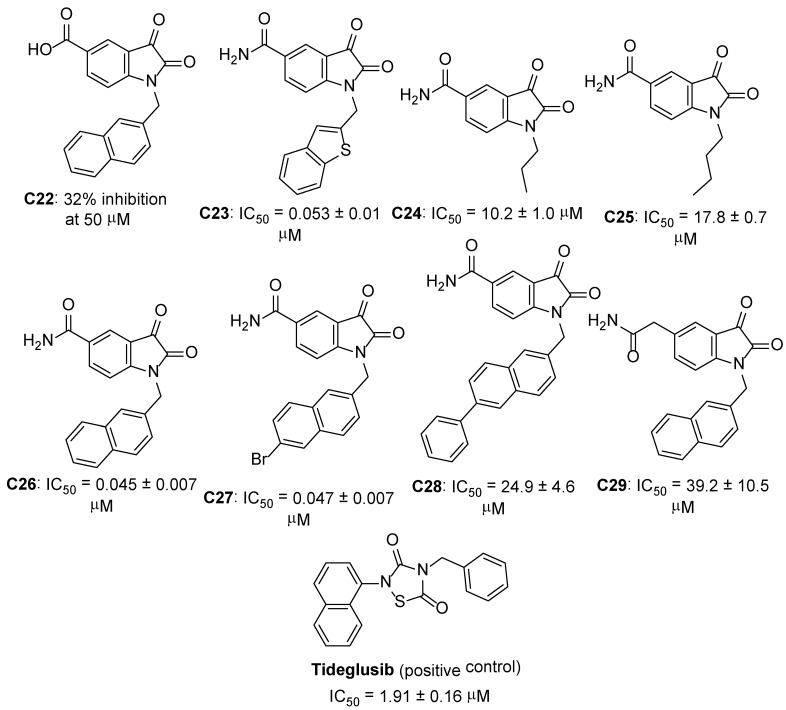
The 3C-like protease (3CL^pro^) inhibitory properties of isatin analogs **13** (**C1–C29**) [143].

A series of isatin derivatives **13** have been synthesized through the reaction of aromatic amines with hydroxylamine hydrochloride (NH_2_OH.HCl) and chloral hydrate [Cl_3_CCH(OH)_2_] followed by cyclization with concentrated sulfuric acid (H_2_SO_4_) at 90 °C and alkylation (Scheme 3). Some of the synthesized isatin analogs **13** (**C1**–**C29**) revealed 3C-like protease (3CL^pro^) [or main protease (M^pro^)] SARS-CoV-2 inhibitory properties relative to Tideglusib (positive control) [143] (Figure 16).

### 4.2. 2-[(Indol-3-yl)thio]acetamides

**Scheme 4:**
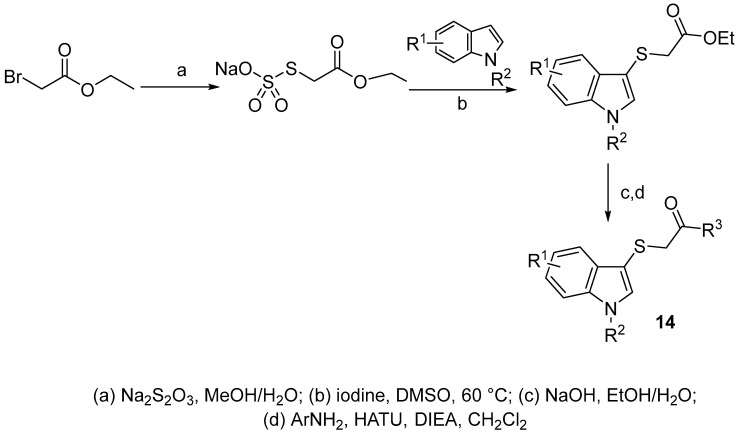
Synthesis of 2-[(indol-3-yl)thio)]acetamides **14** of potential RdRp inhibitory properties [144].

**Figure 17:**
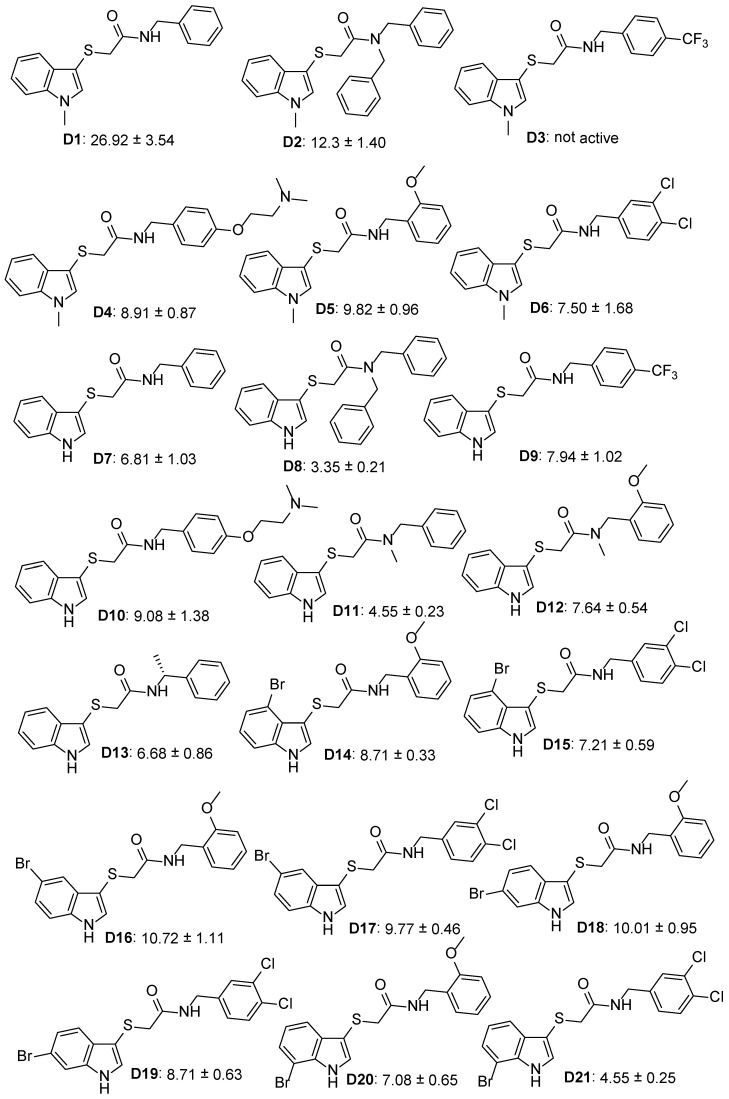

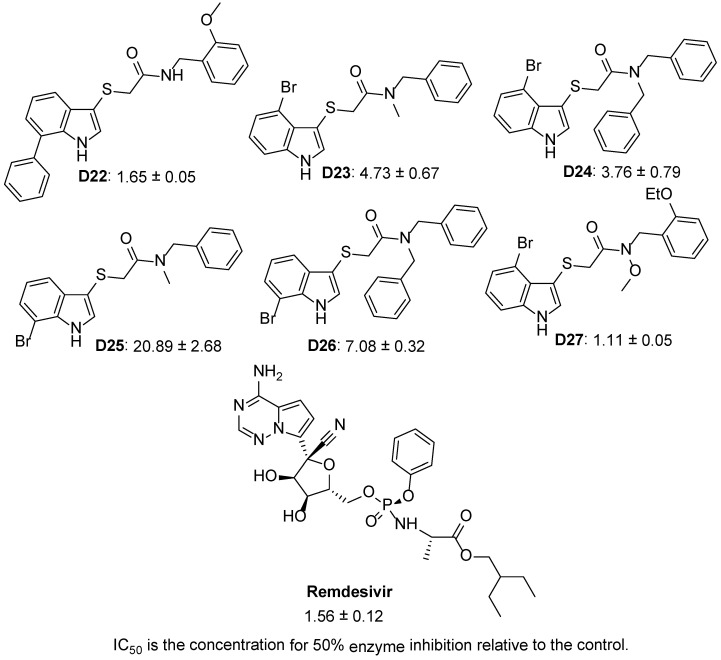
The inhibitory properties of anti-SARSCoV-2 RdRp (IC_50_, µM) of the synthesized 2-[(indol-3-yl)thio)]acetamides **14** (**D1**–**D27**) [144].

2-[(Indol-3-yl)thio]acetamides **14** (**D1**–**D27**) were synthesized through the reaction of indole derivatives with Bunte salt ethyl acetate-2-sodium thiosulfate in iodine/DMSO at 60 °C followed by hydrolysis (NaOH, EtOH/H_2_O) and coupling with the appropriate amine (Scheme 4). Some of the synthesized agents exhibited RdRp inhibitory properties relative to Remdesivir [144] (Figure 17). Considering these observations (Figure 17) and those mentioned in Figure 16, it can be concluded that the substituent attached to the indolyl heterocycle plays a crucial role in the mode of action. Compounds in Figure 16 exhibited 3CL^pro^ inhibitory properties whereas, those of Figure 17 revealed RdRp inhibitory properties. So, the mutual mode of action may be optimized by manipulating the substitution of the indolyl heterocycle for assigning potent anti-SARS-CoV-2 agents.

**Figure 18:**
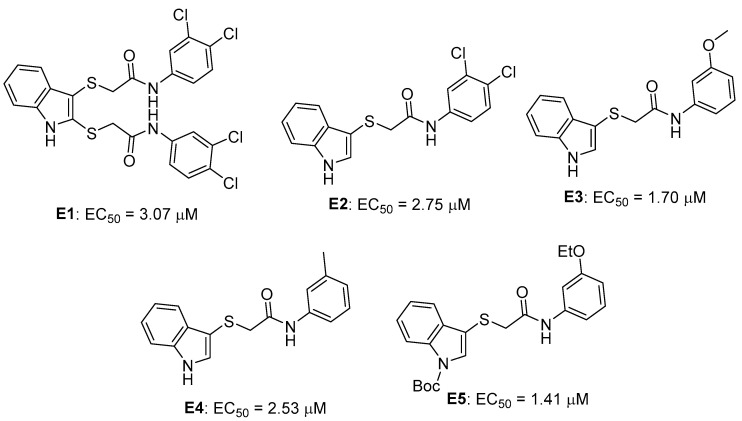
Inhibitory properties of SARS-CoV-2 RdRp (HEK293T cells transfected with nsp12) of acetamide-containing indoles (**E1**–**E5**) [145].

A set of acetamide-containing indoles (**E1**–**E5**) with possible RdRp SARS-CoV-2 inhibitory properties have been explored. The efficacy of the most promising agents was comparable to that of Remdesivir (EC_50_ = 1.05 μM) [145] (Figure 18).

### 4.3. Indole-Chloropyridine Conjugates

**Scheme 5:**
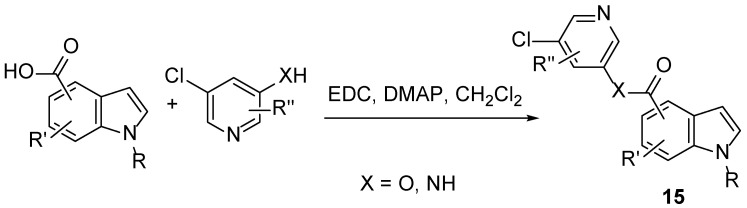
Synthesis of indole-chloropyridine conjugates **15** of potential anti-SARS-CoV-2 properties [59].

**Figure 19:**
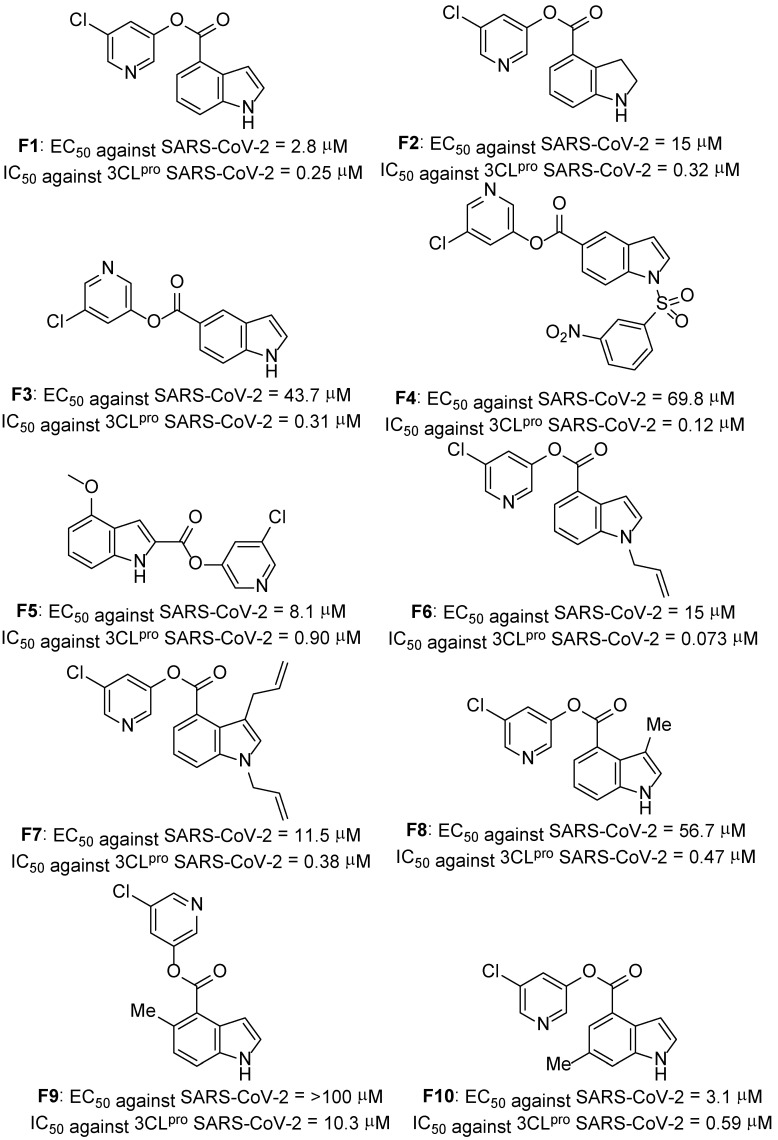

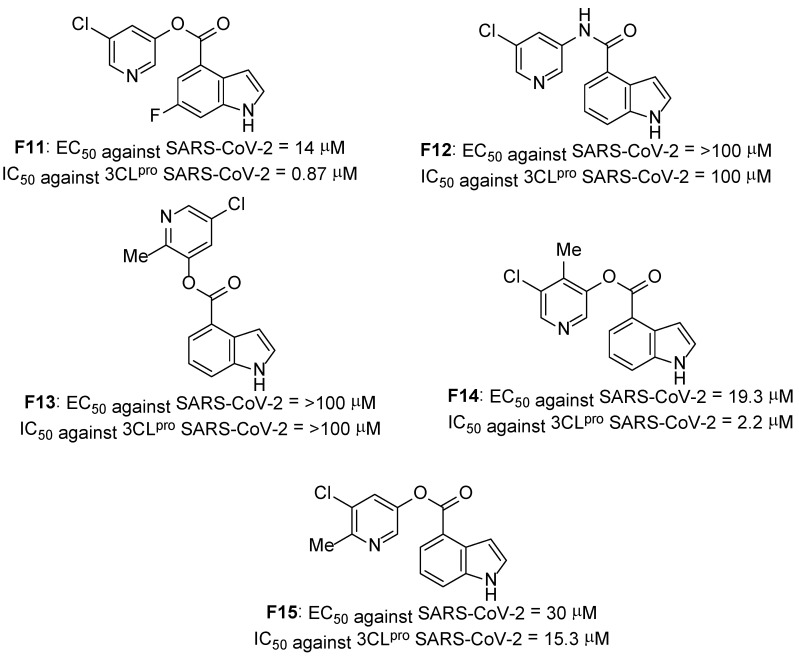
Anti-SARS-CoV-2 and 3CL^pro^ inhibitory properties of the indole-chloropyridine conjugates **15** (**F1**–**F15**) [59].

A variety of indole-chloropyridine conjugates **15** (**F1**–**F15**) were synthesized via reaction of the appropriate indolecarboxylic acid with 3-chloropyridin-5-ols or 5-amino-3-chloropyridines using 1-ethyl-3-(3-dimethylaminopropyl)carbodiimide (EDC) and dimethylaminopyridine (DMAP) in methylene chloride (CH_2_Cl_2_) (Scheme 5). Anti-SARS-CoV-2 activities (Vero E6 assay) with 3CL^pro^ inhibitory properties were observed for some of the synthesized agents [59] (Figure 19).

**Figure 20:**
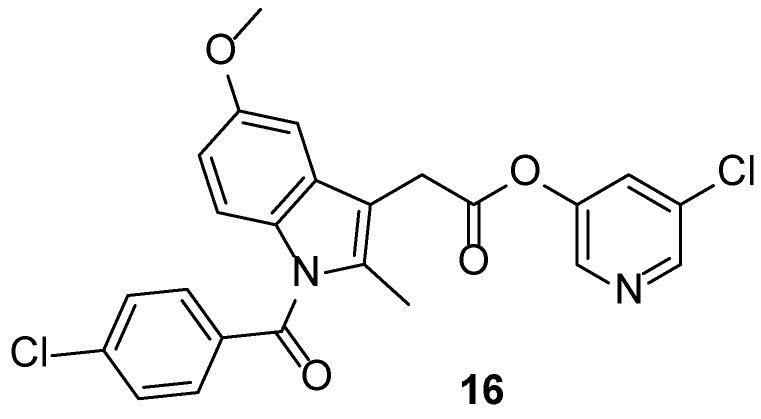
Indomethacin-chloropyridine conjugate **16** of potential anti-SARS-CoV-2 properties [146].

Indomethacin-chloropyridine conjugate **16** (Figure 20) is also possible by utilizing the same reaction conditions (EDC and DMAP in CH_2_Cl_2_, 53% yield). Anti-SARS-CoV-2 activity was observed for the compound (EC_50_ against SARS-CoV-2 = 30.2 μM, IC_50_ against 3CL^pro^ SARS-CoV-2 = 5.32 μM) [146].

### 4.4. Diindole-Substituted Benzimidazole

**Scheme 6:**
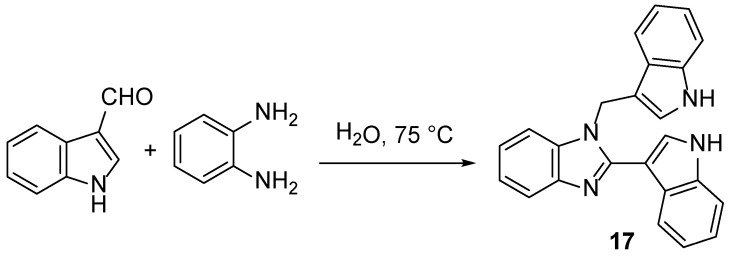
Diindole-substituted benzimidazole **17** [147].

The condensation of 3-indolealdehyde with *o*-phenylenediamine under green conditions (in water at 75 °C) in a 2:1 molar ratio afforded the corresponding diindole-substituted benzimidazole **17** (Scheme 6). The synthesized agent revealed 92.4% cell viability (Vero E6) at 9.0 μM concentration in comparison to 99.23% for Remdesivir at 10 μM. The antiviral properties of **17** were supported by the immunofluorescence assay [147].

### 4.5. 3-Alkenyl-2-Oxindoles

**Scheme 7:**
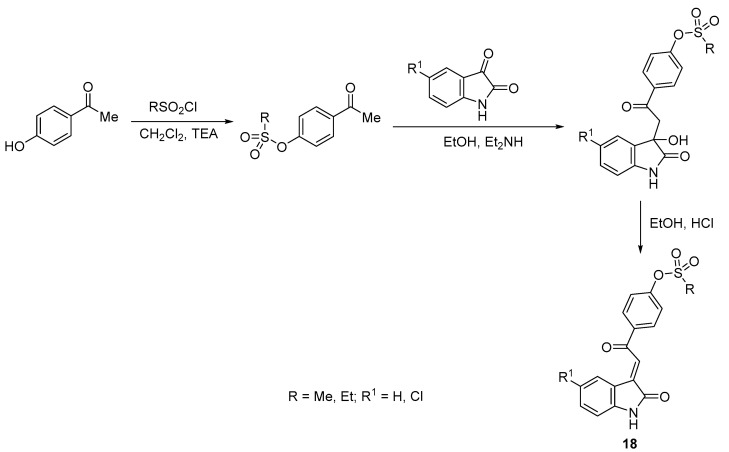
Synthesis of 3-alkenyl-2-oxindoles **18** [148].

**Scheme 8:**
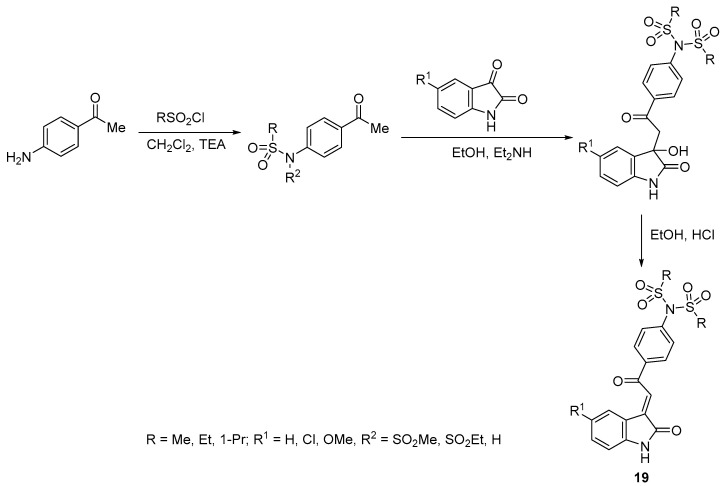
Synthesis of 3-alkenyl-2-oxindoles **19** [148].

**Figure 21:**
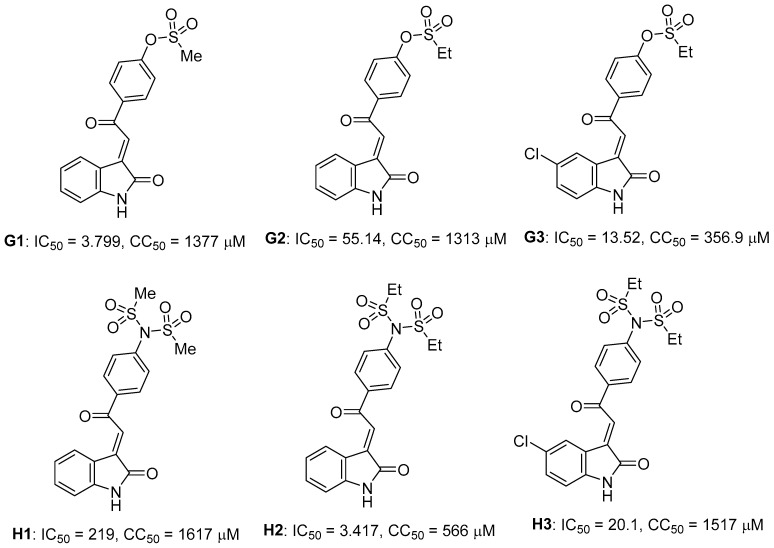
Anti-SARS-CoV-2 properties of tested 3-alkenyl-2-oxindoles **18** (**G1**–**G3**) and **19** (**H1**–**H3**) [148].

Anti-SARS-CoV-2 3-alkenyl-2-oxindoles **18** and **19** were obtained through acidic dehydration (HCl/EtOH) from the corresponding 3-hydroxy analogs (Scheme 7 and Scheme 8). Some of the synthesized agents showed potent anti-SARS-CoV-2 properties (Figure 21) relative to the standards (IC_50_ = 29.25, 19.78, 1382, CC_50_ = 356.4, 377.7, 2633 μM for Hydroxychloroquine, Chloroquine, and Favipiravir, respectively) in the Vero E6 assay [148].

### 4.6. Spiroindoles

Spirocyclic compounds are organic compounds with a rigid, 3D-geometrical structure. In 1911, A. Pictet and T. Spengler reported the first spiro-analog intermediate. Spiroindole-containing compounds are important due to the versatile biological properties established by diverse natural and synthetic analogs originating from the C-3 indolyl ring with many heterocycles affording various motifs.

**Scheme 9:**
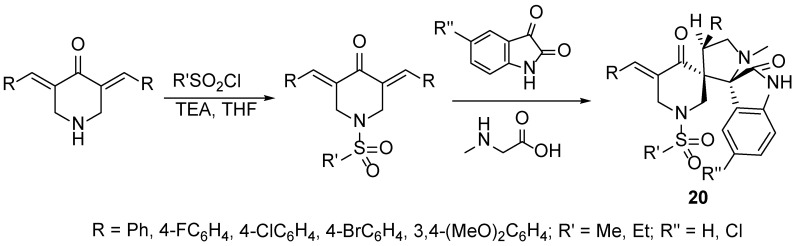
Synthesis of spiroindoles **20** through azomethine ylide cycloaddition [149].

**Figure 22:**
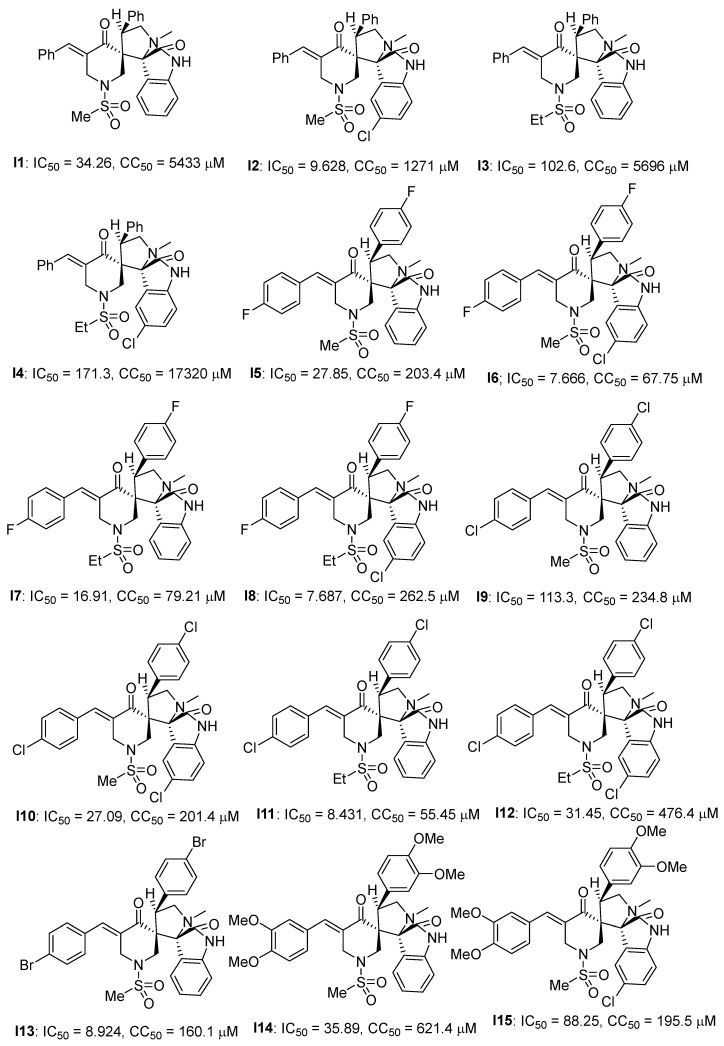
Anti-SARS-CoV-2 properties of spiroindoles **20** (**I1**–**I15**) [149].

Spiroindoles **20** were synthesized through the cycloaddition of azomethine ylide (obtained from sarcosine and isatins) with 3,5-diylidene-4-piperidones (Scheme 9). Promising anti-SARS-CoV-2 properties were shown by some of the synthesized agents (**I1**–**I15**) in the Vero E6 assay relative to the standards Hydroxychloroquine, Chloroquine, and Favipiravir [149] (Figure 22).

**Scheme 10:**
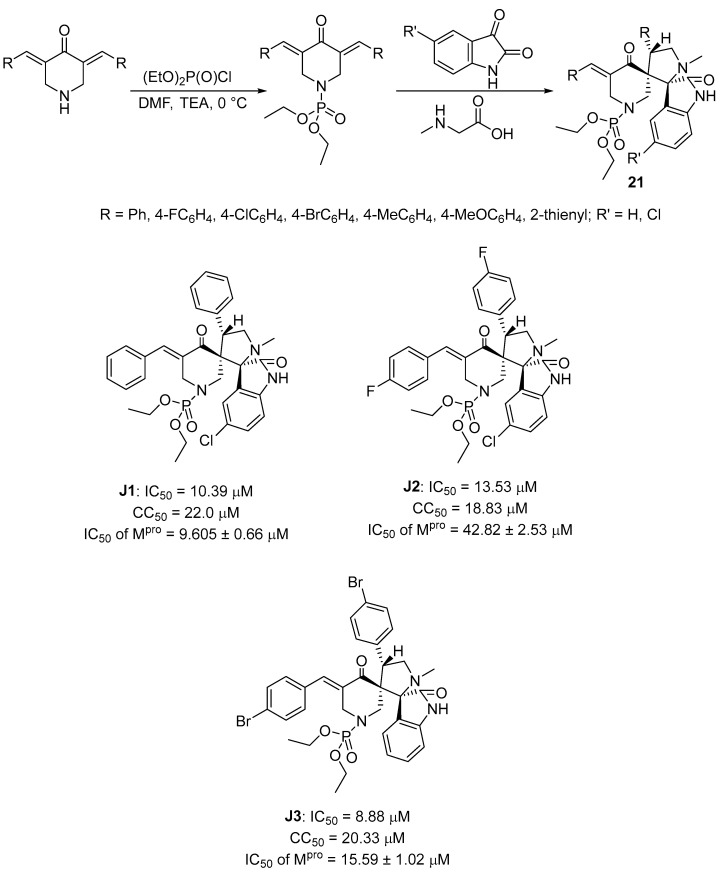
Synthesis of spiroindole-containing compounds **21** (**J1**–**J3**) bearing a phosphonate group [150].

Anti-SARS-CoV-2 spiroindole-containing compounds bearing a phosphonate group **21** (**J1**–**J3**) were recently reported with potential M^pro^ inhibitory properties, synthesized through azomethine (generated from the reaction of isatin and sarcosine) reaction with the appropriate 3,5-bis((*E*)-ylidene)-1-phosphonate-4-piperidone [150] (Scheme 10).

It is notable that the spiroindoles with a sulfonyl group (Figure 22) are more promising anti-SARS-CoV-2 agents relative to those with a phosphonate group.

### 4.7. Indole with Dual Acting Proteases Inhibitor

**Scheme 11:**
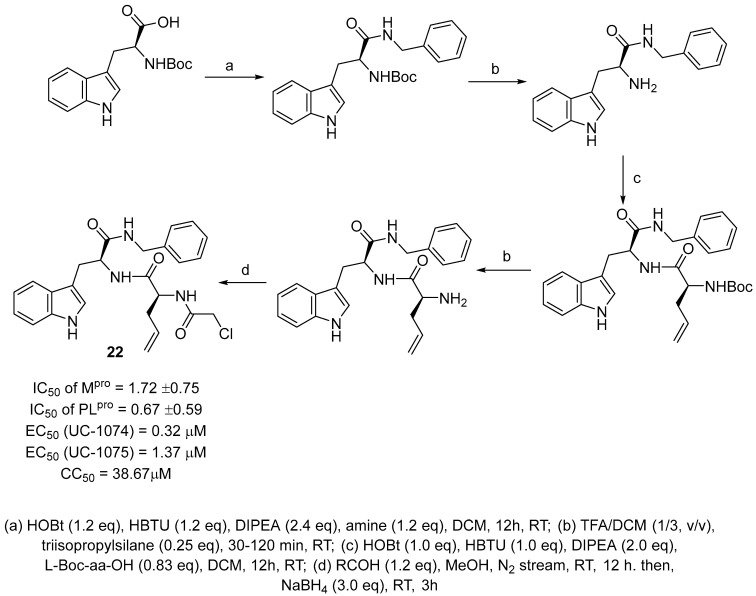
Synthesis of indole-containing compound (**22**) of dual acting protease (M^pro^, PL^pro^) inhibitors [151].

Di Sarno [151] reported the synthesis of an indole-containing compound (**22**) with potential SARS-CoV-2 protease (M^pro^ “main protease” and PL^pro^ “papain-like protease”) inhibitory properties (Scheme 11).

## 5. In Silico Predicted Anti-SARS-CoV-2 Indoles

The use of computational techniques is an accessible approach to identifying effective hits/leads and accelerating the drug discovery program directed towards the development of anti-SARS-CoV-2, either through repurposing or de novo drug design. Virtual screening can reduce the time and cost needed for establishing possible bioactive agents. However, the agents identified by in silico studies still require supporting experimental bio-properties investigations to realize the benefits of these studies [152,153].

### 5.1. SARS-CoV-2 (Main Protease, M^pro^) Inhibitor

SARS-CoV-2 main protease (M^pro^ or 3CL^pro^) controls many essential viral processes including maturation, replication, and transcription. This makes it a potential target for optimizing therapeutics against COVID-19 [154,155]. Paxlovid is a prominent protease inhibitor approved by the FDA at the end of 2021 for mild and moderately effected patients. It is a combination of two therapeutics, Nirmatrelvir (3CL protease inhibitor) and Ritonavir (protease inhibitor, therapeutic against HIV/AIDS). Paxlovid is effective at reducing the hospitalization period when administrated at the beginning of COVID-19 symptoms [156,157,158].

**Scheme 12:**
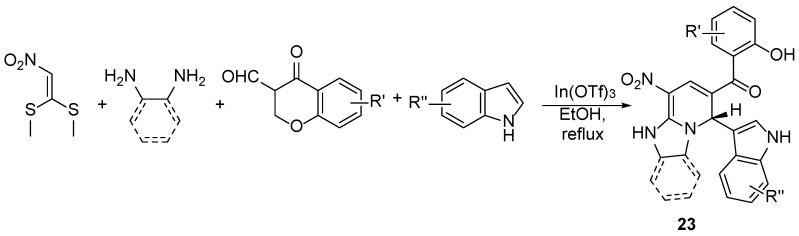
Synthesis of 3-substitued indoles **23** [62].

**Figure 23:**
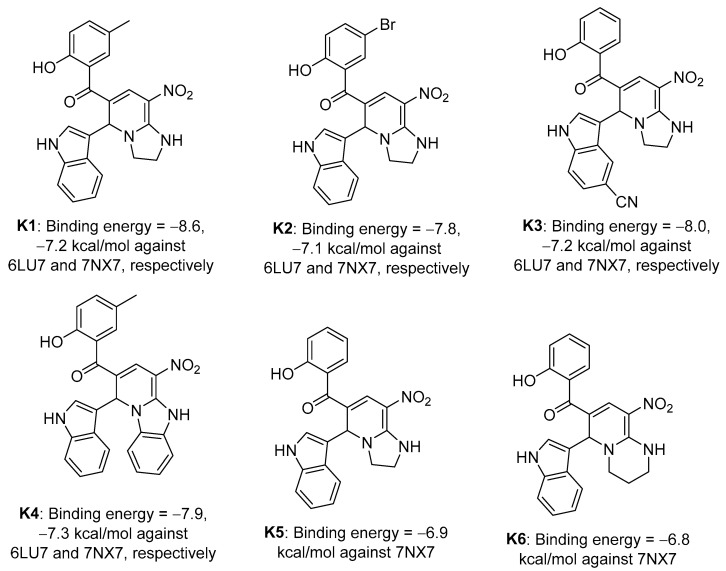
In silico predicted 3-substituted indoles **23** (**K1**–**K6**) of potential inhibitory properties against main protease 6LU7 and spike glycoprotein 7NX7 [62].

**Figure 24:**
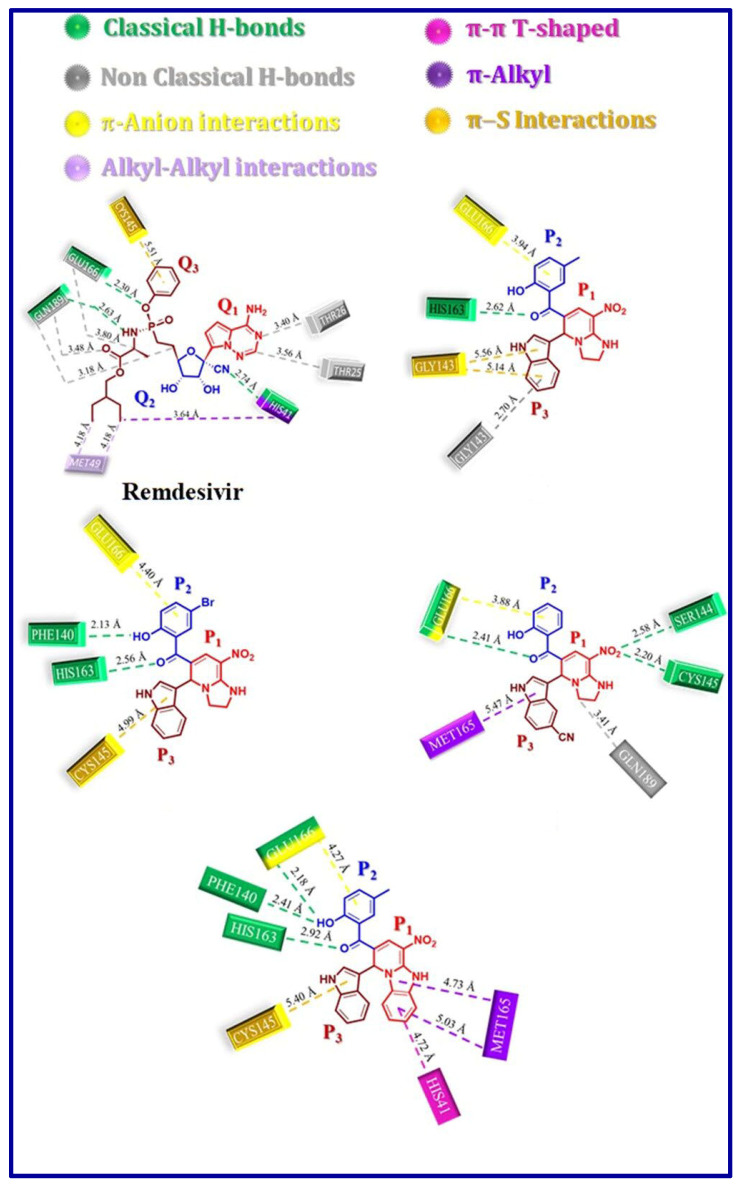

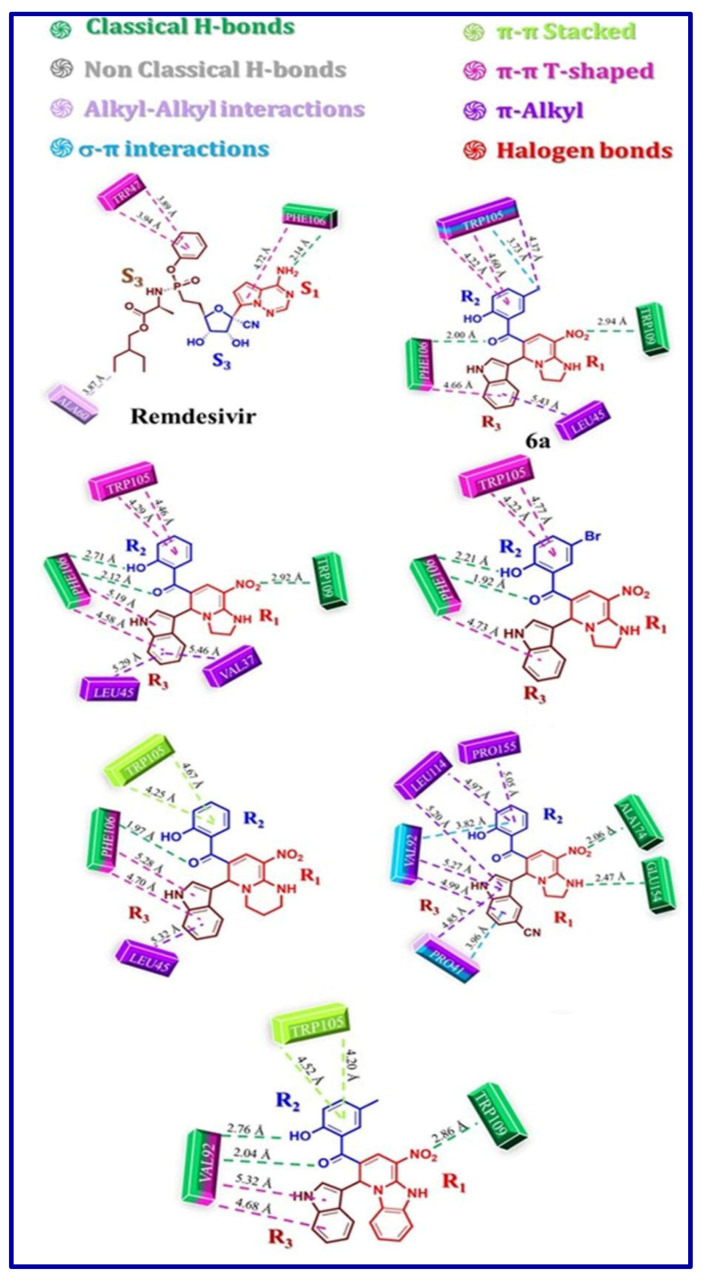
(**upper**) Representation of Remdesivir and predicted 3-substituted indoles **23** revealing potential properties against 6LU7 [62]. (**lower**) Representation of Remdesivir and predicted 3-substituted indoles **23** revealing potential properties against 7NX7 [62].

In this section, representative examples of computationally predicted M^pro^ SARS-CoV-2 inhibitors will be highlighted. Jayabal et al. reported the synthesis of 3-substituted indoles **23** through a multi-component green synthetic approach via the reaction of nitroketene *S*,*S*-acetal, diamine-containing compound, 3-formylchromone, and indole in the presence of In(OTf)_3_ as a catalyst in refluxing ethanol [62] (Scheme 12). Computationally, some of the synthesized agents (**K1**–**K6**) showed inhibitory properties for SARS-COV-2 M^pro^ (PDB: 6LU7) and spike glycoprotein (PDB: 7NX7) utilizing Auto Dock-Vina software (v. 1.1.2). For comparison, Remdesivir binding energy = −7.7, −6.5 kcal/mol is against main protease 6LU7 and spike glycoprotein 7NX7, respectively [62] (Figure 23 and Figure 24).

**Figure 25:**
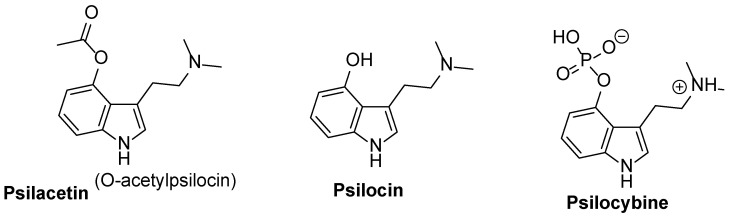
Psilacetin, psilocin, and psilocybine of potential anti-M^pro^ SARS-CoV-2 [159].

**Figure 26:**
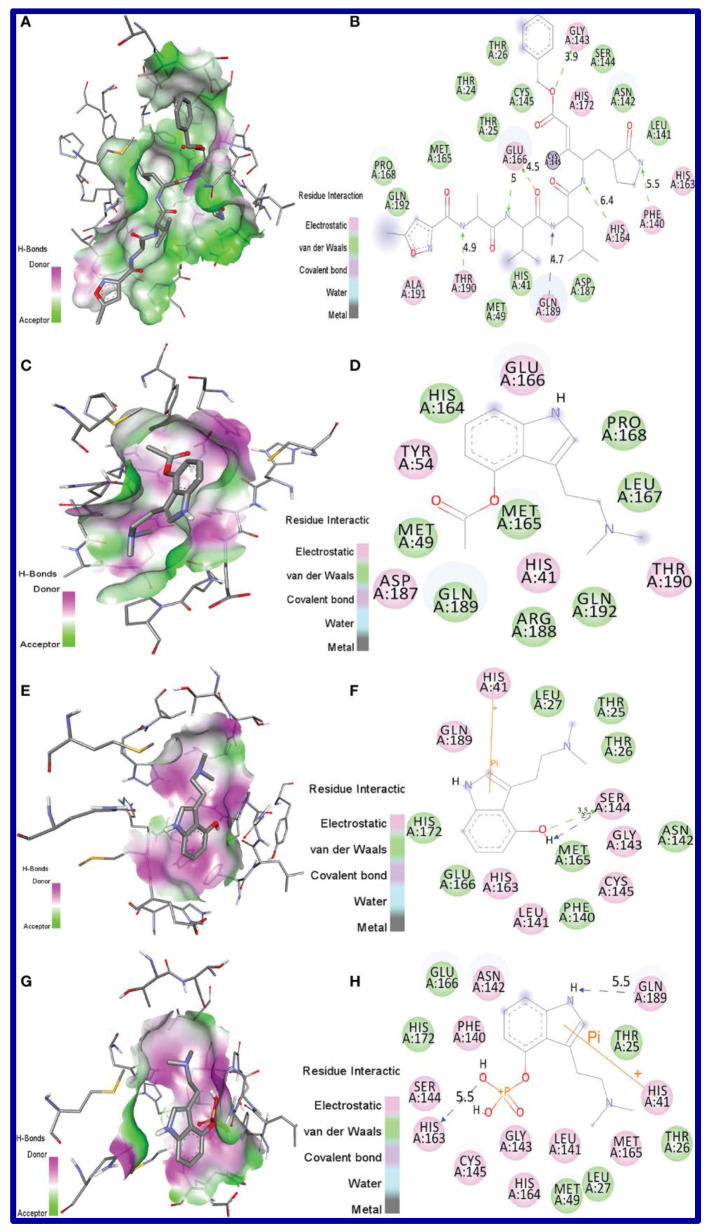
2D- and 3D-docking interactions of (**A**,**B**), N3 inhibitor; (**C**,**D**), psilacetin; (**E**,**F**), psilocin; and (**G**,**H**), psilocybine in the active pocket of M^pro^ SARS-CoV-2 (PDB: 6LU7) [159].

Many mushroom metabolites have potential biological activities. Psilacetin, psilocin, and psilocybine, which are psilocybin-mushroom components, have been subjected to M^pro^ SARS-CoV-2 docking studies (PDB: 6LU7) utilizing AutoDock and AutoDock vina software. They reveal considerable binding affinity in the protein active site (interaction docking scores = −6.0, −5.4, −5.8 kcal/mol for psilacetin, psilocin, and psilocybine, respectively) [159] (Figure 25 and Figure 26).

**Figure 27:**
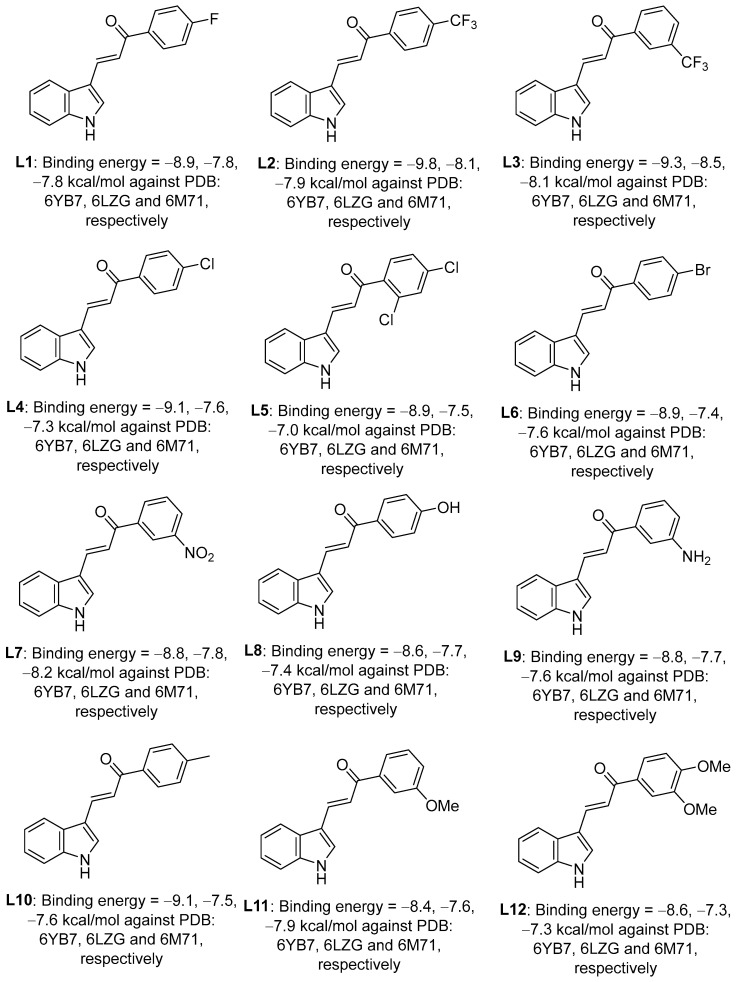

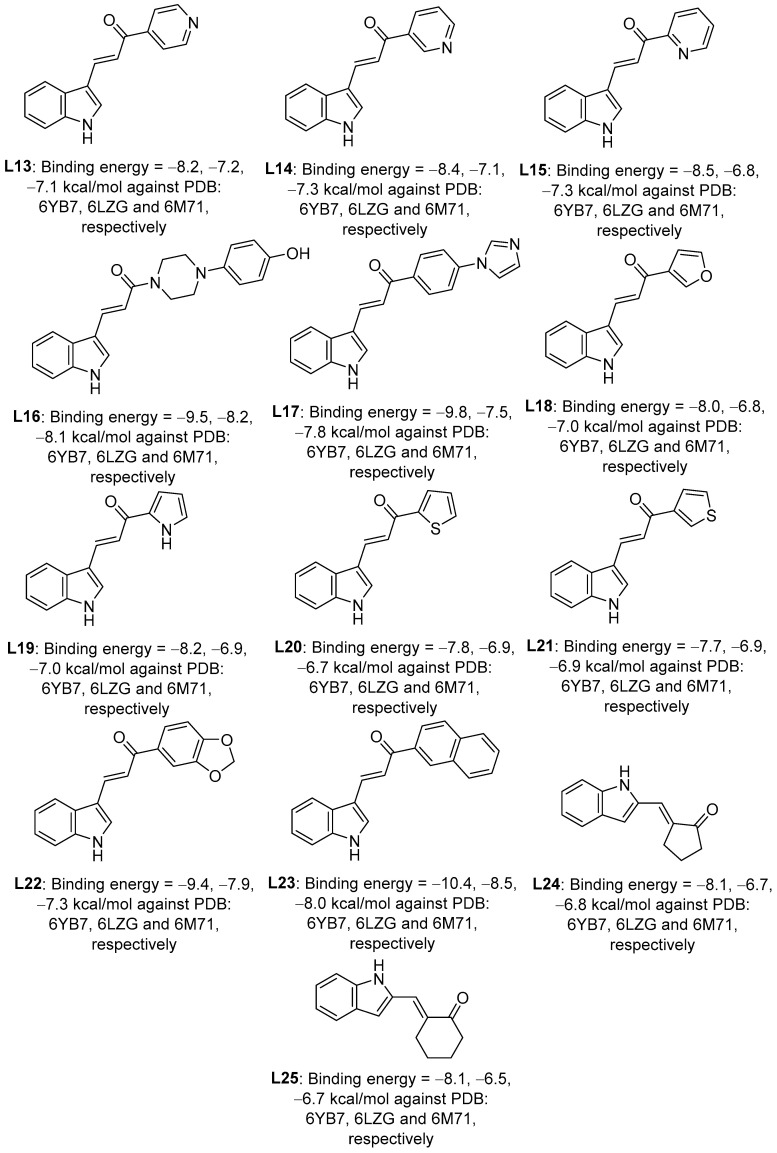
In silico predicted indolyl chalcones (**L1**–**L25**) and their binding energies in M^pro^ (PDB: 6YB7), spike protein (PDB: 6LZG), and RNA-dependent RNA polymerase (PDB: 6M71) [160].

A series of indolyl chalcones (**L1**–**L25**) have also been explored against M^pro^ (PDB: 6YB7), spike protein (PDB: 6LZG), and RNA-dependent RNA polymerase (PDB: 6M71) in silico by the blind docking technique utilizing AutoDock Vina v.1.1.2. Some of the results suggested promising inhibitory properties that may help narrow the search for anti-SARS-CoV-2 candidates [160] (Figure 27).

**Scheme 13:**
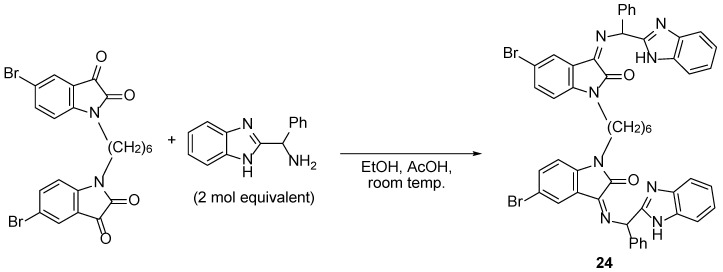
Synthesis of bis-Schiff base **24** [161].

**Figure 28:**
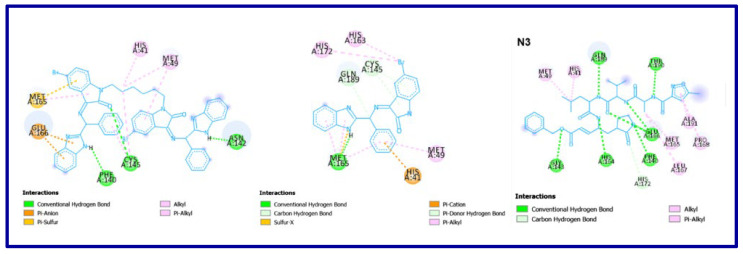
2D-docking interaction of Schiff bases and N3 in the active site of M^pro^ SARS-CoV-2 (PDB: 6LU7) [161].

The Schiff bases formed from the condensation of isatin and 2-(1-aminobenzyl)benzimidazole revealed in silico possible M^pro^ SARS-CoV-2 inhibitory properties (3CL protease, PDB: 6LU7, AutoDock 4.2 software). The 5-bromo-substituted analog of bis-Schiff base **24** formed from the condensation of 2-(1-aminobenzyl)benzimidazole and the corresponding bis-isatin in ethanol containing a few drops of AcOH at room temperature (Scheme 13) is the most promising [161] (Figure 28).

**Figure 29:**
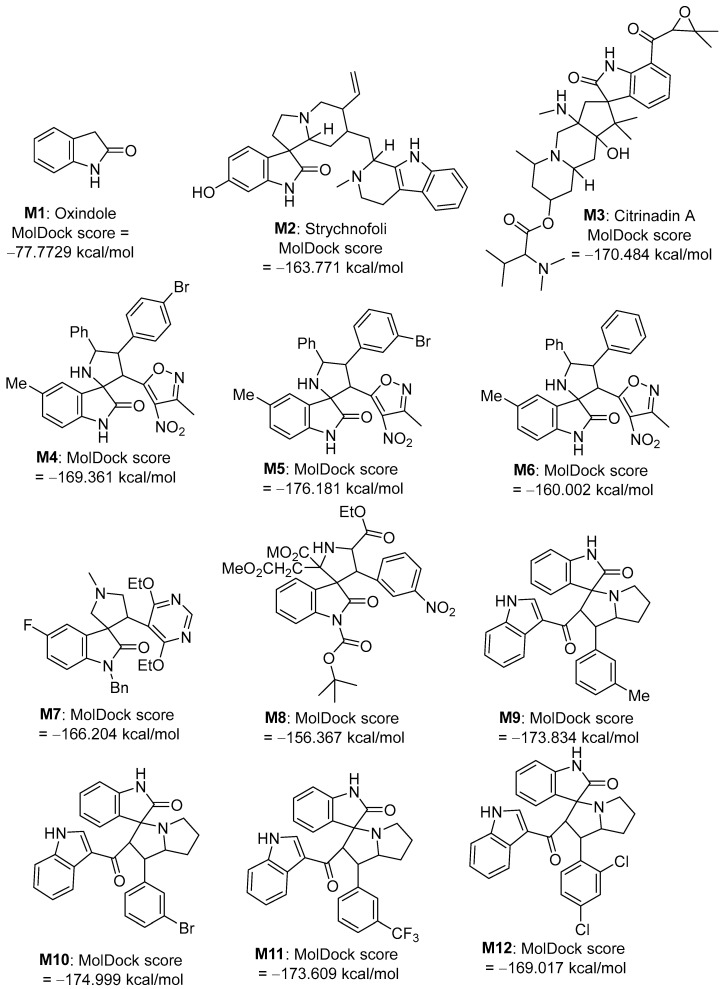

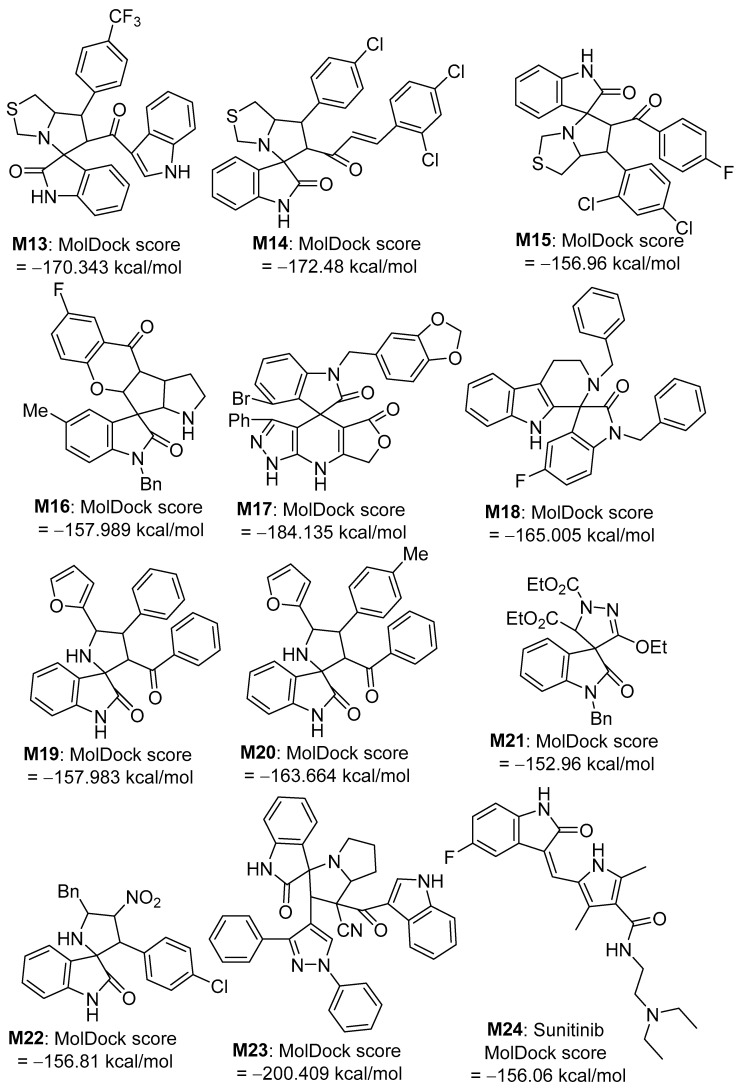

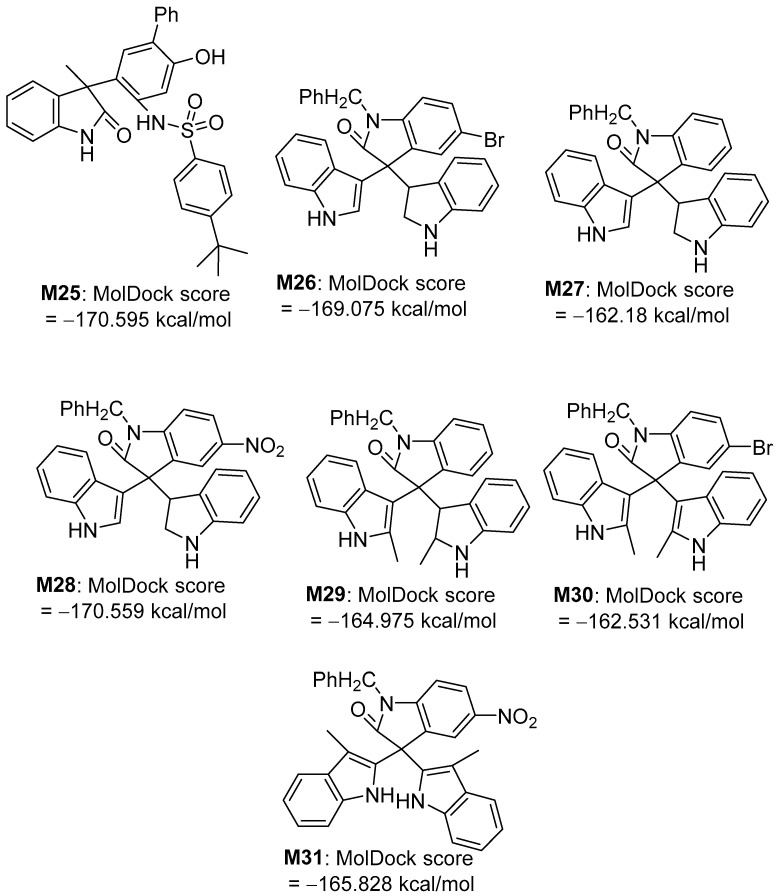
Oxindoles (**M1**–**M31**) with in silico M^pro^ SARS-CoV-2 properties [162].

A set of 2-oxindole derivatives (**M1**–**M31**) with in silico M^pro^ SARS-CoV-2 inhibitory properties were mentioned (PDB ID: 6LU7, Molegro Virtual Docker version 7.0.0 Software, MVD) [162] (Figure 29).

**Figure 30:**
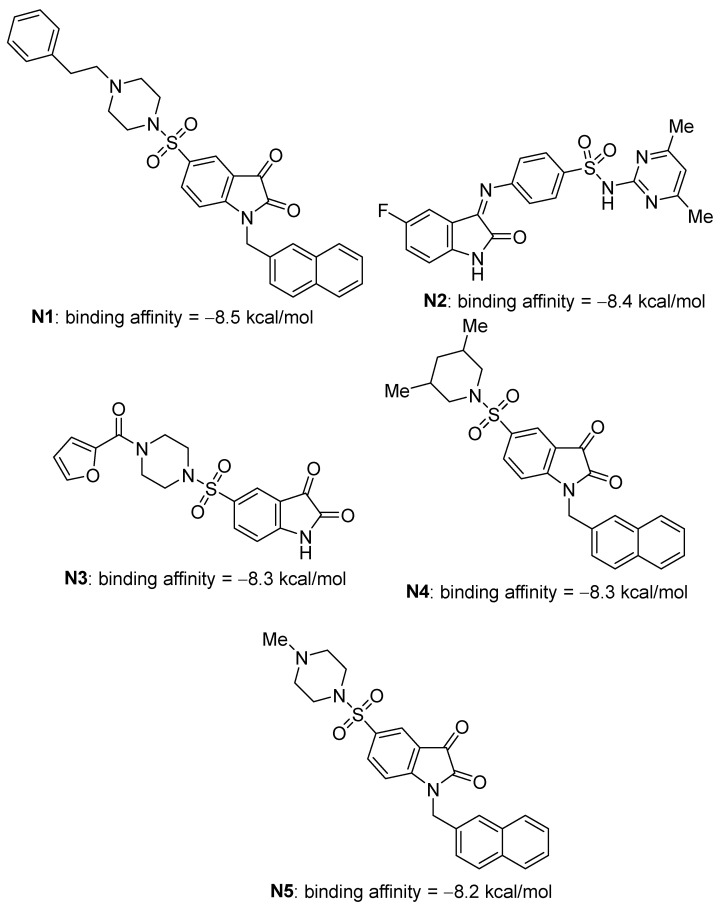
Isatin analogs (**N1**–**N5**) with potenial in silico M^pro^ SARS-CoV-2 inhibitory properties [72].

**Figure 31:**
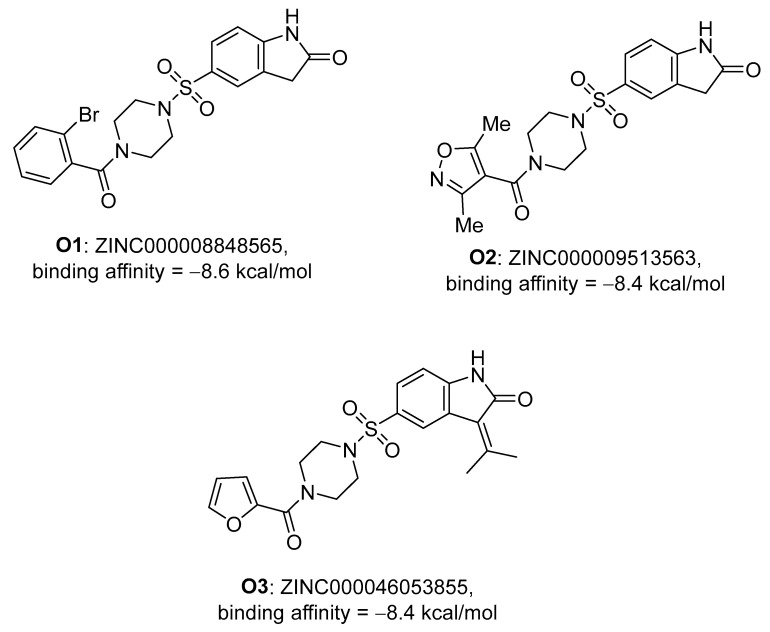
Indole analogs (**O1**–**O3**) through Zinc drug-like library with potential properties against M^pro^ SARS-CoV-2.

A set of isatin-based protease inhibitors was collected from previous publications followed by in silico high throughput screening in the active pocket of M^pro^ SARS-CoV-2 (chain-A, PDB: 6M03). The most promising agents (**N1**–**N5**) were identified based on the observed binding affinities (Figure 30). Searching the Zinc drug-like library for similar analogs followed by virtual screening (AutoDock Vina) identified indole analogs (**O1**–**O3**) with potential inhibitory properties against M^pro^ SARS-CoV-2 [72] (Figure 31).

**Figure 32:**
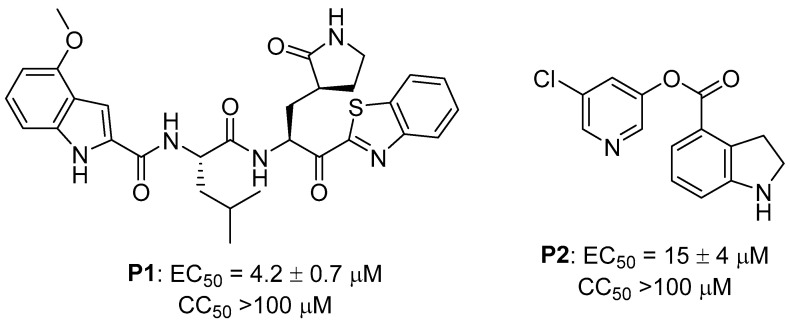
Indole-containing compounds (**P1**, **P2**) of potential in vitro anti-SARS-CoV-2 and in silico M^pro^ properties [163].

Hattori et al. have also reported the in silico M^pro^ SARS-CoV-2 properties (PDB: 6Y2F, Maestro Version 10.7.015) in addition to the in vitro activity (Vero E6 assay) of indole-containing compounds (**P1** and **P2**) [163] (Figure 32).

**Scheme 14:**
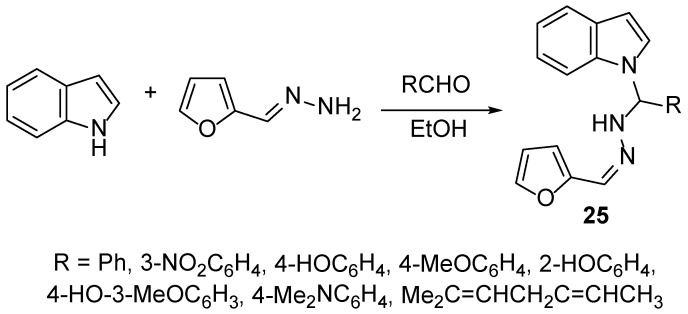
Synthesis of indole-containing compounds **25** [164].

**Figure 33:**
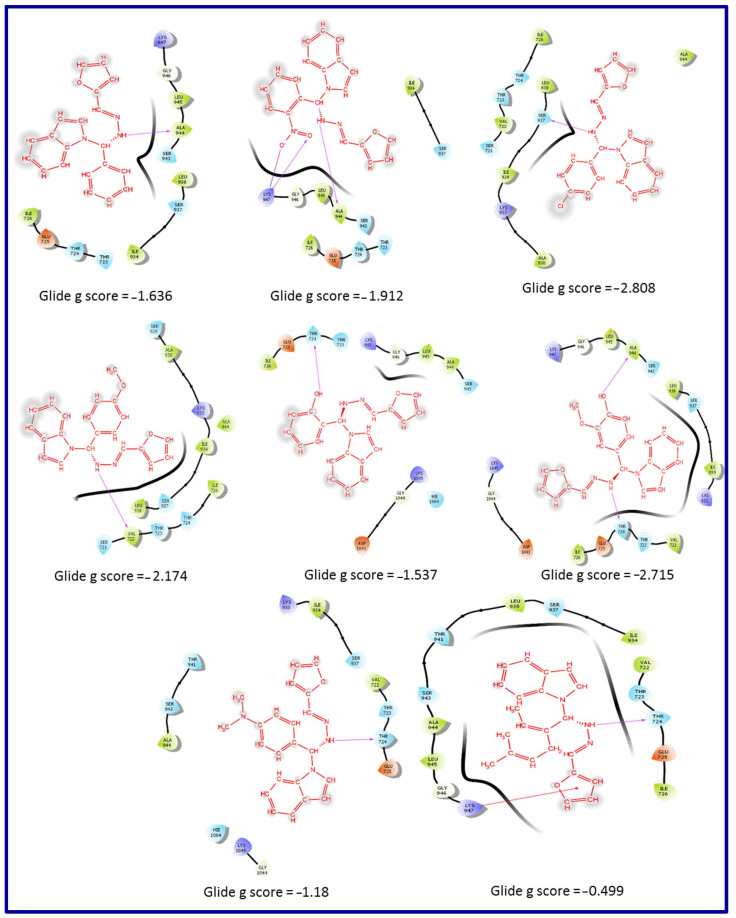
2D-docking interaction of indole-containing compounds **25** in the active site of SARS-CoV-2 spike glycoprotein (PDB: 6WPT) [164].

2-Indole-containing compounds **25** were obtained through the reaction of indole, furan-2-ylmethylenehydrazine, and appropriate aldehyde in ethanol (Scheme 14). Considerable activity against SARS-CoV-2 spike glycoprotein (PDB: 6WPT, Schrodinger 12.4 software) was observed for some of the synthesized agents [164] (Figure 33).

**Figure 34:**
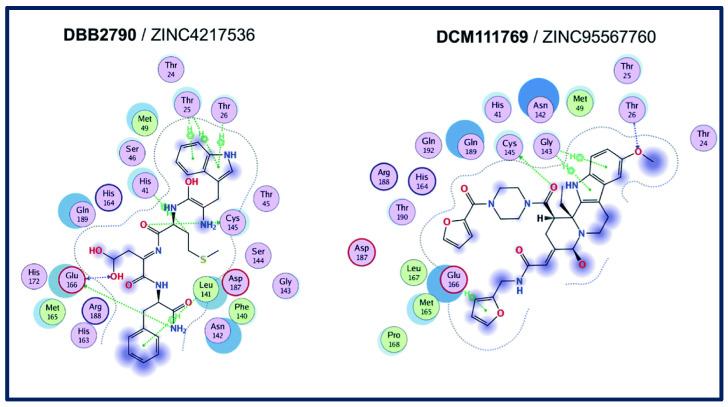
2D-docking interaction of Compounds ID: ZINC4217536 and ZINC95567760 in PDB ID 6LU7 [165].

A computational study considered food chemicals and components named as dark chemical matters could predict some effective anti-SARS-CoV-2 hits. Compound ID: ZINC4217536 (ZINC database) was mentioned as a promising antiviral active agent due to docking observations in M^pro^ of SARS-CoV-2 (PDB ID 6LU7) utilizing MOE (Molecular Operating Environment v.2019 software). It reveals hydrogen bonding interaction with CYS145 and GLU166 in addition to a π-interaction with HIS41 (all these amino acids are the key components of the protein active site). Compound ID: ZINC95567760, which contains a fused indolyl heterocycle, also reveals a promising docking interaction in the PDB: 6LU7. Hydrogen bonding interaction with CYS145, in addition to π-interactions with GLU166 and GLY143, support these assumptions [165] (Figure 34).

### 5.2. RdRp (RNA-Dependent RNA Polymerase) Inhibitor

The RdRp enzyme is one of the most reliable targets for optimizing potent antiviral therapeutics. This is attributed to its ability to terminate the viral RNA replication in addition to the lack of any similar RdRp in the host cell, thus minimizing off-target effects [9,166].

**Figure 35:**
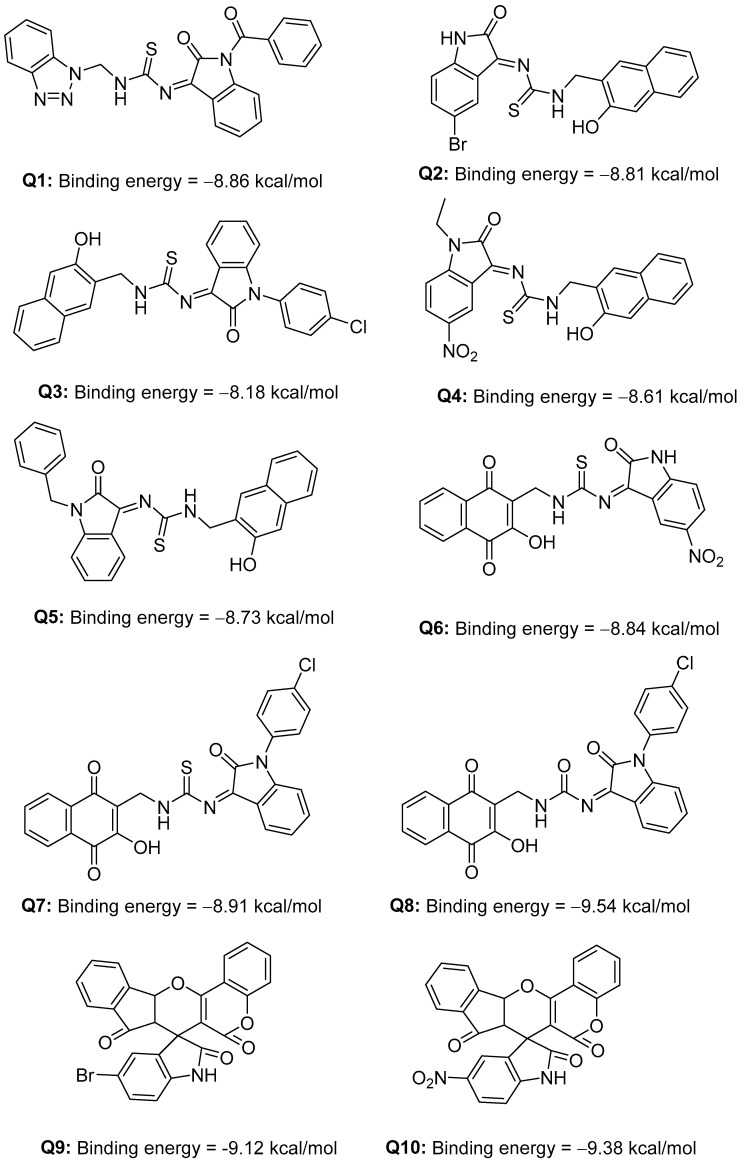
Binding energy of potential RdRp isatin analogs (**Q1**–**Q10**) in PDB ID: 7BTF active pocket [167].

A computational study has also explored isatin analogs (**Q1**–**Q10**) for the identification of promising RdRp SARS-CoV-2 inhibitor agents (PDB ID: 7BTF, AutoDock); the most promising compounds discovered are exhibited in Figure 35 [167].

López-López et al. [168] have pointed out that an analysis of ChEMBL (chemical database of bioactive agents created by the European Bioinformatics Institute) indicates that 10 μM is a convenient benchmark by which to differentiate active from inactive compounds. Assigning such parameters can help distinguish between active and inactive compounds as well as help improve effectiveness. Structure activity/property relationship (SAR/APR) software can assist with this aspect. Manipulating the chemical structure based on the physic-chemical parameters (descriptors) can turn the inactive or mildly active agents into potent effective ones. This explains the interest of medicinal chemistry researchers in QSAR/QSPR studies [119,169,170].

## 6. Conclusions

COVID-19 has proven to be one of the most serious crises facing human health in recorded history. The scientific community has been tirelessly working to optimize effective therapeutics. While vaccination has been successful in controlling the pandemic, research into the effective treatment of current and future mutants remains crucial. One area of focus has been on the indole scaffold, which includes many alkaloid categories and has shown promise in the fight against COVID-19. Repurposed indole-containing drugs, as well as various natural and synthetic indole analogs, have been investigated for anti-SARS-CoV-2 efficacy. In silico studies were utilized to generate new hits and optimize leads against SARS-CoV-2. 3-Alkenyl-2-oxindoles and spiroindoles are potentially valuable anti-SARS-CoV-2 agents that can be synthesized in a regio-selective approach. From the cited reports, it can be concluded that indole-containing compounds are important lead molecules and can be further optimized for the development of potential agents against SARS-CoV-2.

## Supplementary Materials

The following supporting information can be downloaded at: https://www.mdpi.com/article/10.3390/molecules28186603/s1, Table S1: List of potential Indole-based compounds and their activities (IC50/EC50) against SARS-CoV-2.

## Sample Availability

Not application.
