# Supplementary material for: Indole-Based Compounds as Potential Drug Candidates for SARS-CoV-2
Source: Molecules. 2023 Sep 13;28(18):6603. doi: 10.3390/molecules28186603 (PMC10537473; doi:10.3390/molecules28186603)
Supplement: Supplementary file 1 [file molecules-28-06603-s001.zip › molecules-2571198-supplementary.pdf]

Supporting Information

Indole-based compounds as potential drug candidates for SARS-CoV-2

Adel S. Girgis *[a], Siva S. Panda *[b], Benson M. Kariuki [c], Mohamed S. Bekheit [a], Reham F. Barghash [a], Dalia R. Aboshouk [a]

[a] *Department of Pesticide Chemistry, National Research Centre, Dokki, Giza, 12622, Egypt*

[b] *Department of Chemistry and Biochemistry, Augusta University, Augusta, GA 30912, USA*

[c] *School of Chemistry, Cardiff University, Main Building, Park Place, Cardiff, CF10 3AT, UK*

* Corresponding authors: sspanda12@gmail.com or sipanda@augusta.edu (S.S. Panda) and as.girgis@nrc.sci.eg or girgisas10@yahoo.com (A.S. Girgis)

Table S1: List of potential Indole-based compounds and their activities (IC50/EC50) against SARS-CoV-2

S.No.	Compound Name	Compound Structure	IC_{50}/EC_{50}	Virus/Enzyme
1	**Arbidol**	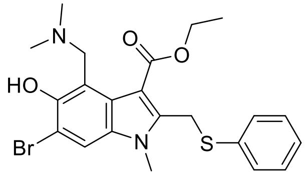	IC_{50} = 4.11 μM	SARS-CoV-2
2	**Indomethacin**	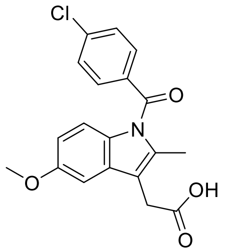	EC_{50} = 94.9 μM	SARS-CoV 2/NL/2020

3	**B1**	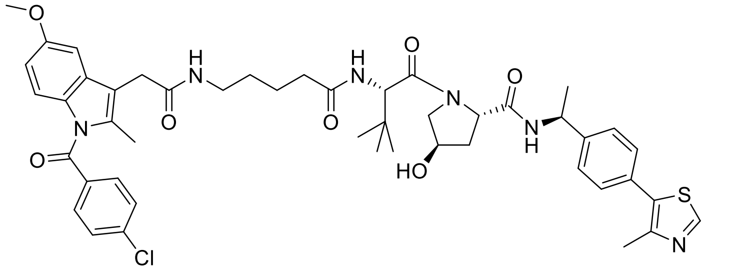	EC_{50} = >50 µM<br>EC_{50} = >50 µM	SARS-CoV 2/NL/2020<br>SARS-CoV-2/Padova/2021
4	**B2**	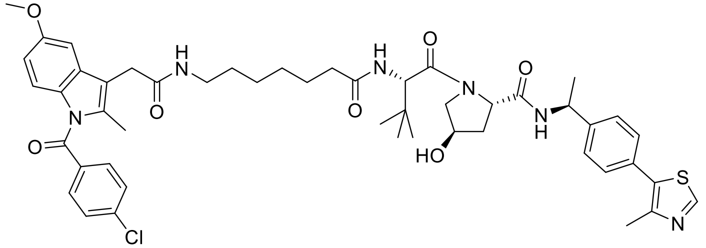	EC_{50} = 18.1 µM<br>EC_{50} = 25.4 µM	SARS-CoV 2/NL/2020<br>SARS-CoV-2/Padova/2021
5	**B3**	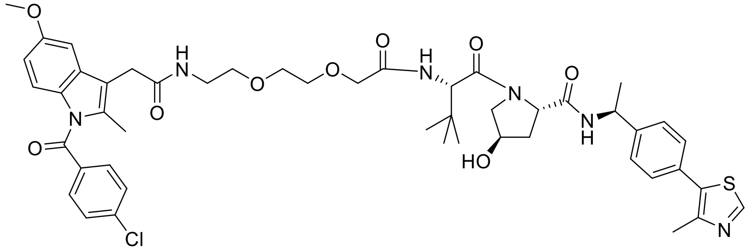	EC_{50} = >50 µM<br>EC_{50} = >50 µM	SARS-CoV 2/NL/2020<br>SARS-CoV-2/Padova/2021
6	**Lufotrelvir (PF-07304814)**	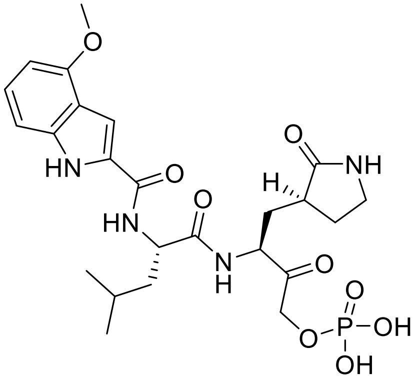	IC_{50} = 0.692 µM	M^{pro} SARSCoV-2

7	**PF-00835231**	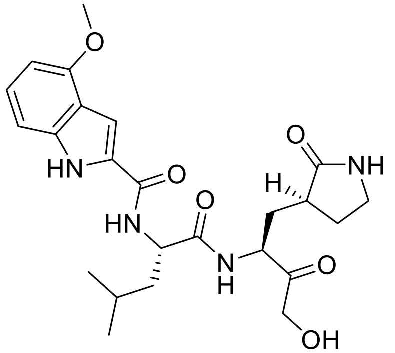	IC_{50} = 0.009 μM	M^{pro} SARSCoV-2
8	**Obatoclax (GX15-070)**	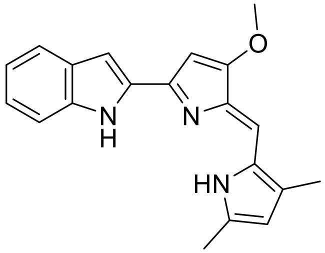	EC_{50}: 23.2 μM	Nijmegen1
9	**Neoechinulin A**	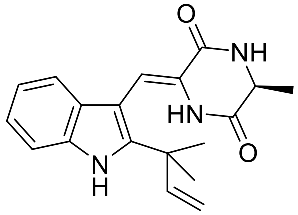	IC_{50} = 0.47 μM	M^{pro}-SARS-CoV-2
10	**Echinulin**	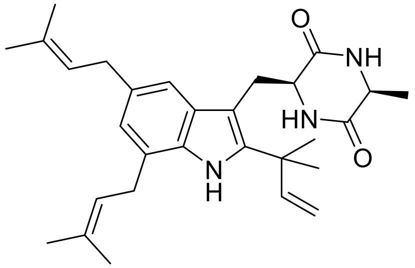	IC_{50} = 3.90 μM	M^{pro}-SARS-CoV-2

11	**GC376**	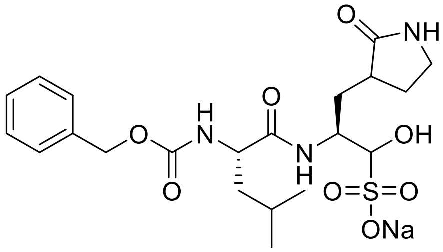	IC_{50} = 0.36 µM	M^{pro}-SARS-CoV-2
12	**Neoechinulin B**	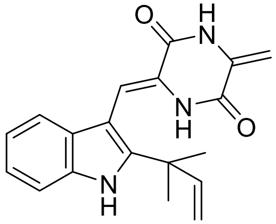	IC_{50} = 32.9 µM	Vero E6
13	**C9**	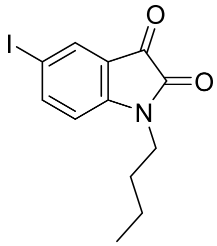	IC_{50} = 41.8 ± 8.0 µM	M^{pro}-SARS-CoV-2
14	**C17**	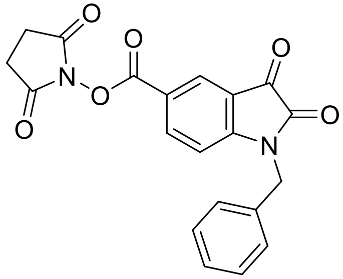	IC_{50} = 15.5 ± 1.2 µM	M^{pro}-SARS-CoV-2

15	**C23**	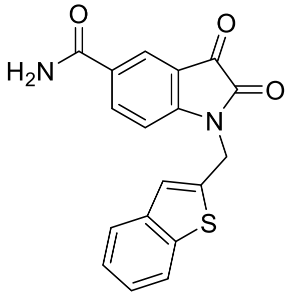	IC_{50} = 0.053 ± 0.01 μM	M^{pro}-SARS-CoV-2
16	**C24**	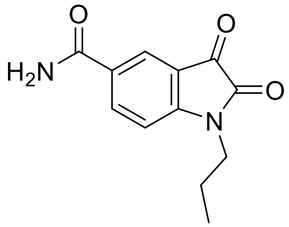	IC_{50} = 10.2 ± 1.0 μM	M^{pro}-SARS-CoV-2
17	**C25**	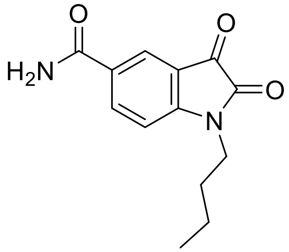	IC_{50} = 17.8 ± 0.7 μM	M^{pro}-SARS-CoV-2
18	**C26**	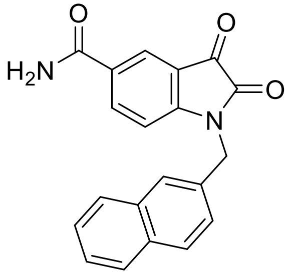	IC_{50} = 0.045 ± 0.007 μM	M^{pro}-SARS-CoV-2

19	**C27**	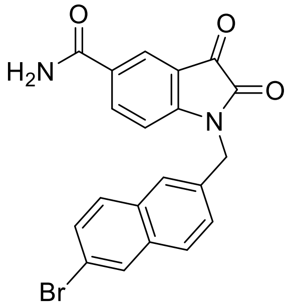	IC_{50} = 0.047 ± 0.007 μM	SARS-CoV-2 RdRp
20	**C28**	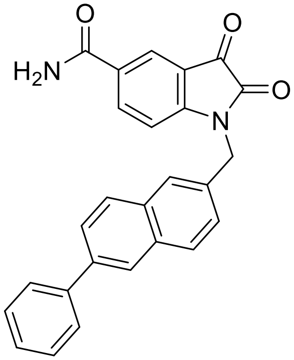	IC_{50} = 24.9 ± 4.6 μM	SARS-CoV-2 RdRp
21	**C29**	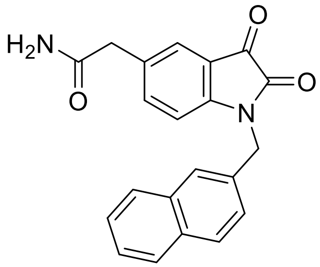	IC_{50} = 39.2 ± 10.5 μM	SARS-CoV-2 RdRp
22	**Tideglusib**	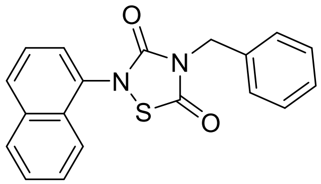	IC_{50} = 1.91 ± 0.16 μM	SARS-CoV-2 RdRp

23	**D1**	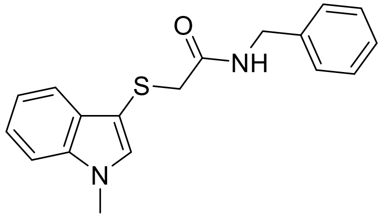	IC_{50} = 26.92 ± 3.54 μM	SARS-CoV-2 RdRp
24	**D2**	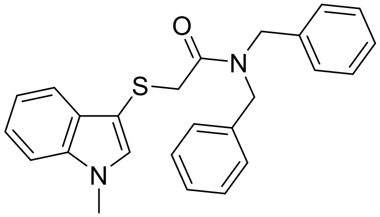	IC_{50} = 12.3 ± 1.40 μM	SARS-CoV-2 RdRp
25	**D3**	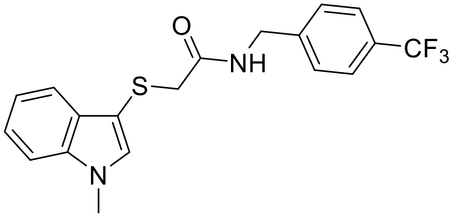	Not active	SARS-CoV-2 RdRp
26	**D4**	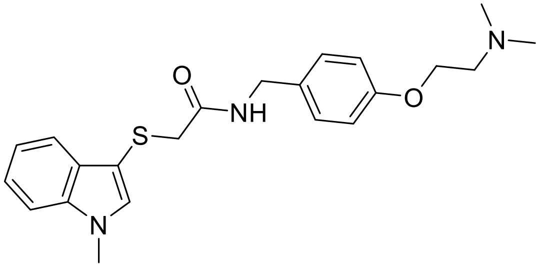	IC_{50} = 8.91 ± 0.87 μM	SARS-CoV-2 RdRp
27	**D5**	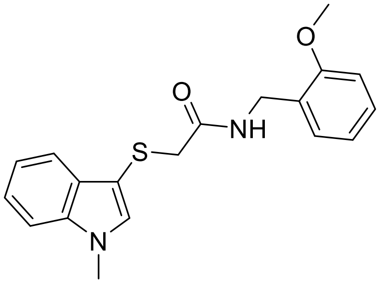	IC_{50} = 9.82 ± 0.96 μM	SARS-CoV-2 RdRp

28	**D6**	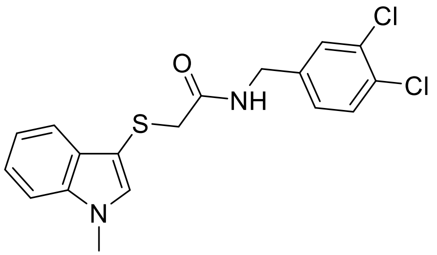	IC_{50} = 7.50 ± 1.68 μM	SARS-CoV-2 RdRp
29	**D7**	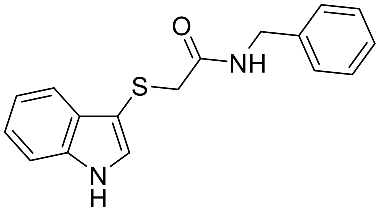	IC_{50} = 6.81 ± 1.03 μM	SARS-CoV-2 RdRp
30	**D8**	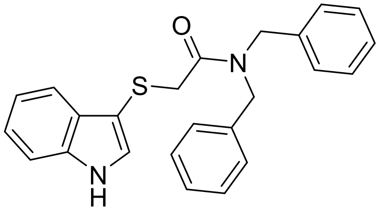	IC_{50} = 3.35 ± 0.21 μM	SARS-CoV-2 RdRp
31	**D9**	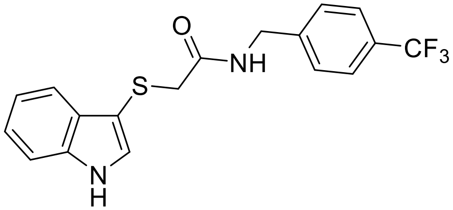	IC_{50} = 7.94 ± 1.02 μM	SARS-CoV-2 RdRp
32	**D10**	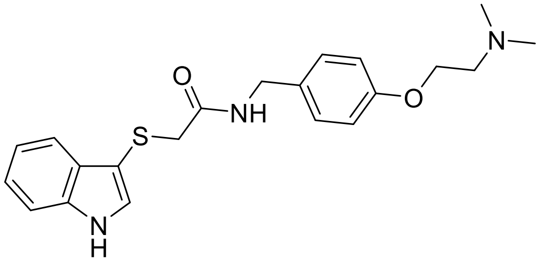	IC_{50} = 9.08 ± 1.38 μM	SARS-CoV-2 RdRp

33	**D11**	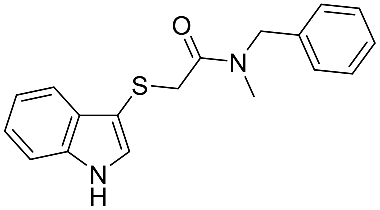	IC$_{50}$ = 4.55 ± 0.23 μM	SARS-CoV-2 RdRp
34	**D12**	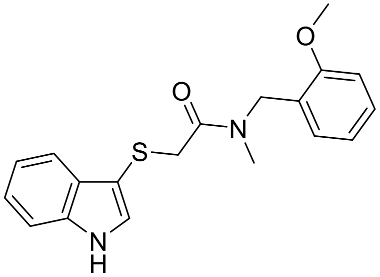	IC$_{50}$ = 7.64 ± 0.54 μM	SARS-CoV-2 RdRp
35	**D13**	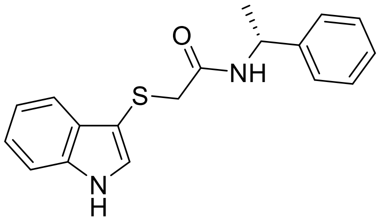	IC$_{50}$ = 6.68 ± 0.86 μM	SARS-CoV-2 RdRp
36	**D14**	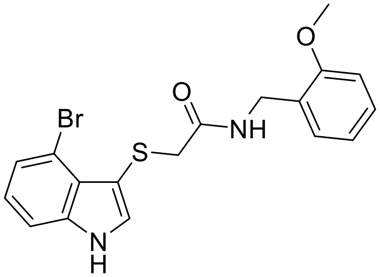	IC$_{50}$ = 8.71 ± 0.33 μM	SARS-CoV-2 RdRp

37	**D25**	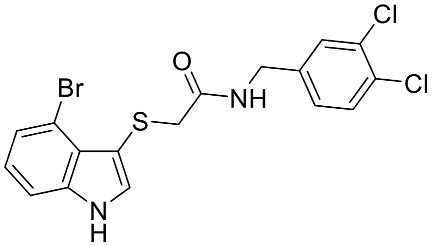	IC_{50} = 7.21 ± 0.59 μM	SARS-CoV-2 RdRp
38	**D16**	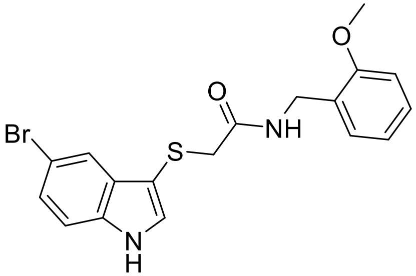	IC_{50} = 10.72 ± 1.11 μM	SARS-CoV-2 RdRp
39	**D17**	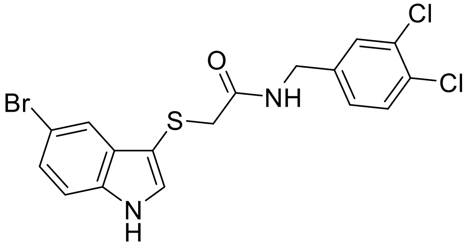	IC_{50} = 9.77 ± 0.46 μM	SARS-CoV-2 RdRp
40	**D18**	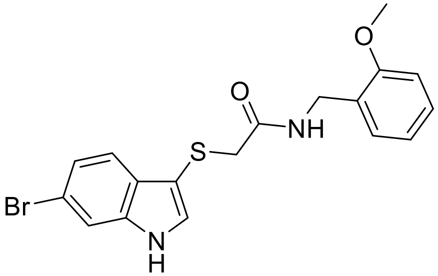	IC_{50} = 10.01 ± 0.95 μM	SARS-CoV-2 RdRp

41	**D19**	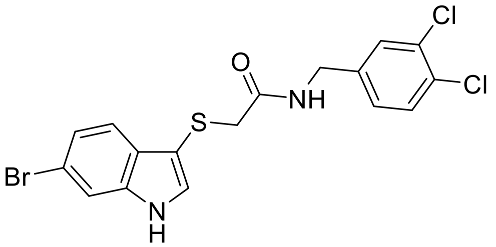	IC_{50} = 8.71 ± 0.63 μM	SARS-CoV-2 RdRp
42	**D20**	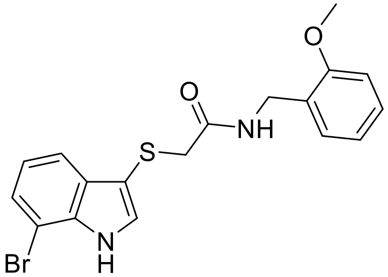	IC_{50} = 7.08 ± 0.65 μM	SARS-CoV-2 RdRp
43	**D21**	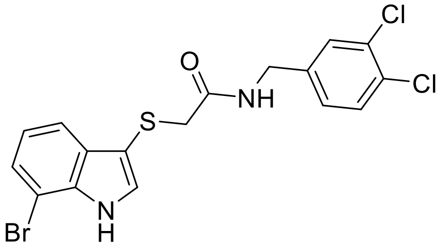	IC_{50} = 4.55 ± 0.25 μM	SARS-CoV-2 RdRp
44	**D22**	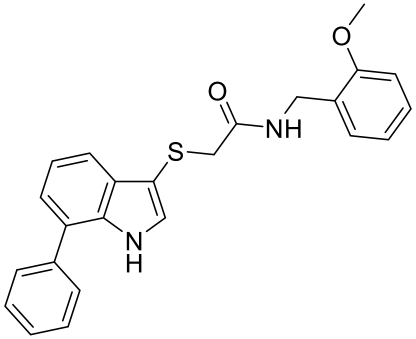	IC_{50} = 1.65 ± 0.05 μM	SARS-CoV-2 RdRp

45	**D23**	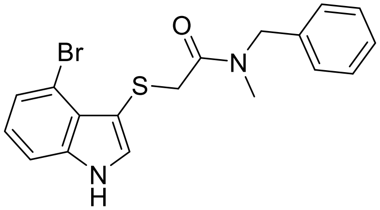	IC_{50} = 4.73 ± 0.67 μM	SARS-CoV-2 RdRp
46	**D24**	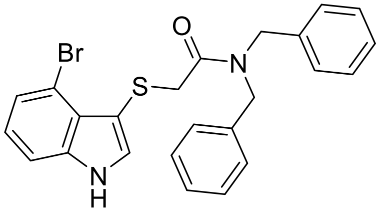	IC_{50} = 3.76 ± 0.79 μM	SARS-CoV-2 RdRp
47	**D25**	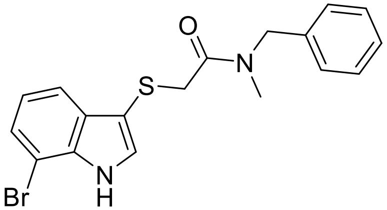	IC_{50} = 20.89 ± 2.68 μM	SARS-CoV-2 RdRp
48	**D26**	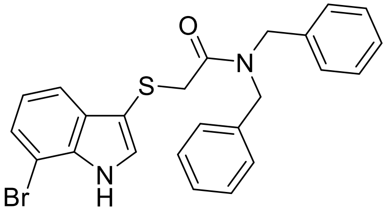	IC_{50} = 7.08 ± 0.32 μM	SARS-CoV-2 RdRp
49	**D27**	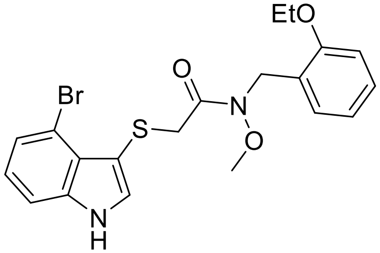	IC_{50} = 1.11 ± 0.05 μM	SARS-CoV-2 RdRp

50	**Remdesivir**	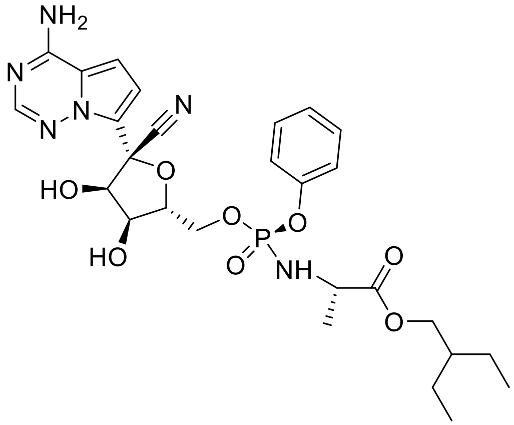	IC_{50} = 1.56 ± 0.12 μM<br>EC_{50} = 1.05 μM	SARS-CoV-2 RdRp
51	**E1**	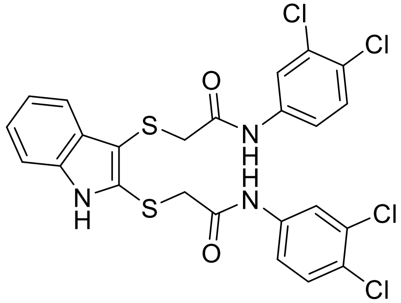	EC_{50} = 3.07 μM	SARS-CoV-2 RdRp
52	**E2**	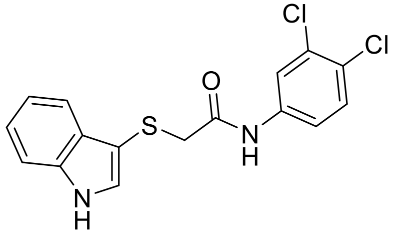	EC_{50} = 2.75 μM	SARS-CoV-2 RdRp
53	**E3**	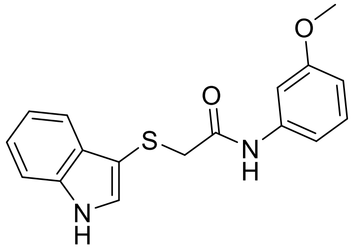	EC_{50} = 1.70 μM	SARS-CoV-2 RdRp

54	**E4**	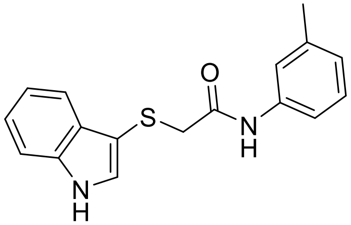	EC_{50} = 2.53 μM	SARS-CoV-2 RdRp
55	**E5**	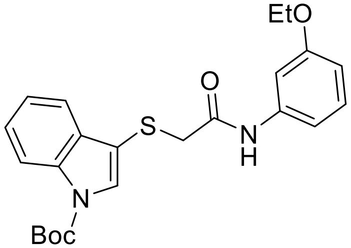	EC_{50} = 1.41 μM	SARS-CoV-2 RdRp
56	**F1**	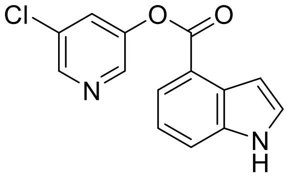	EC_{50} = 2.8 μM<br>IC_{50} = 0.25 μM	SARS-CoV-2<br>M^{pro}-SARS-CoV-2
57	**F2**	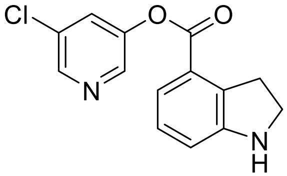	EC_{50} = 15 μM<br>IC_{50} = 0.32 μM	SARS-CoV-2<br>M^{pro}-SARS-CoV-2
58	**F3**	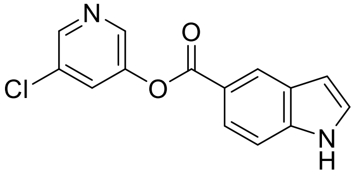	EC_{50} = 43.7 μM<br>IC_{50} = 0.31 μM	SARS-CoV-2<br>M^{pro}-SARS-CoV-2

59	**F4**	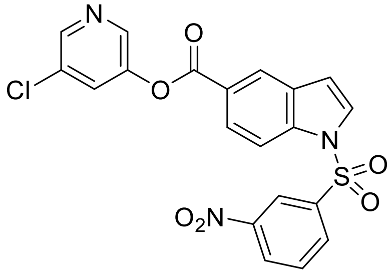	EC_{50} = 69.8 μM<br>IC_{50} = 0.12 μM	SARS-CoV-2<br>M^{pro}-SARS-CoV-2
60	**F5**	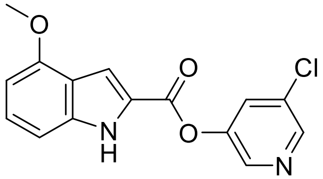	EC_{50} = 8.1 μM<br>IC_{50} = 0.90 μM	SARS-CoV-2<br>M^{pro}-SARS-CoV-2
61	**F6**	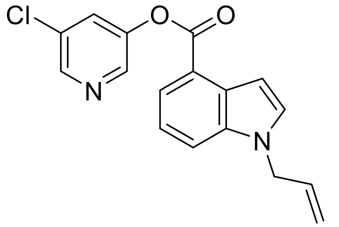	EC_{50} = 15 μM<br>IC_{50} = 0.073 μM	SARS-CoV-2<br>M^{pro}-SARS-CoV-2
62	**F7**	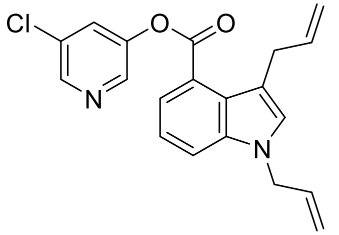	EC_{50} = 11.5 μM<br>IC_{50} = 0.38 μM	SARS-CoV-2<br>M^{pro}-SARS-CoV-2
63	**F8**	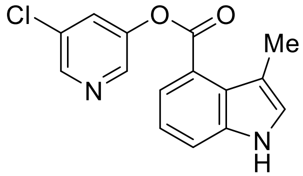	EC_{50} = 56 μM<br>IC_{50} = 0.47 μM	SARS-CoV-2<br>M^{pro}-SARS-CoV-2

64	**F9**	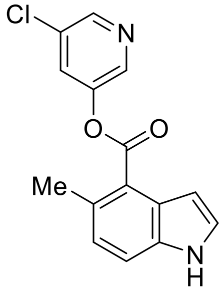	EC_{50} = >100 µM<br>IC_{50} = 10.3 µM	SARS-CoV-2<br>M^{pro}-SARS-CoV-2
65	**F10**	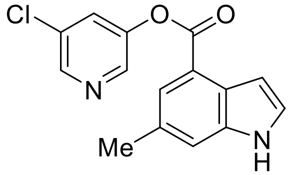	EC_{50} = 3.1 µM<br>IC_{50} = 0.59 µM	SARS-CoV-2<br>M^{pro}-SARS-CoV-2
66	**F11**	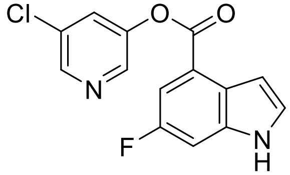	EC_{50} = 14 µM<br>IC_{50} = 0.87 µM	SARS-CoV-2<br>M^{pro}-SARS-CoV-2
67	**F12**	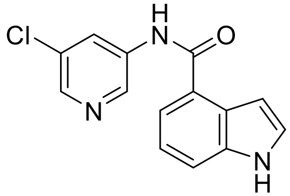	EC_{50} = >100 µM<br>IC_{50} = 100 µM	SARS-CoV-2<br>M^{pro}-SARS-CoV-2
68	**F13**	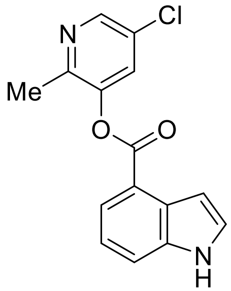	EC_{50} = >100 µM<br>IC_{50} = >100 µM	SARS-CoV-2<br>M^{pro}-SARS-CoV-2

69	**F14**	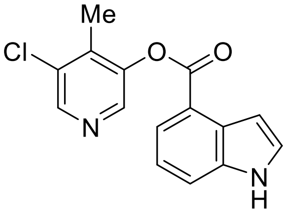	EC_{50} = 19.3 μM<br>IC_{50} = 2.2 μM	SARS-CoV-2<br>M^{pro}-SARS-CoV-2
70	**F15**	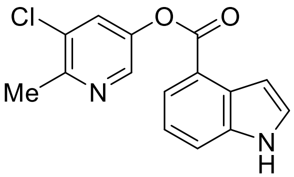	EC_{50} = 30 μM<br>IC_{50} = 15.3 μM	SARS-CoV-2<br>M^{pro}-SARS-CoV-2
71	**16**	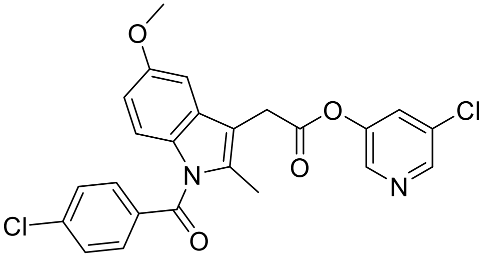	EC_{50} = 30.2 μM<br>IC_{50} = 5.32 μM	SARS-CoV-2<br>M^{pro}-SARS-CoV-2
72	**Hydroxychloroquine**	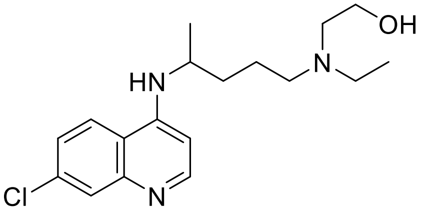	IC_{50} = 29.25 μM	Vero E6
73	**Chloroquine**	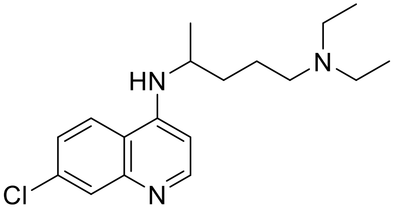	IC_{50} = 19.78 μM	Vero E6
74	**Favipiravir**	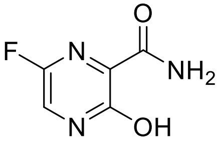	IC_{50} = 1382 μM	Vero E6

75	**G1**	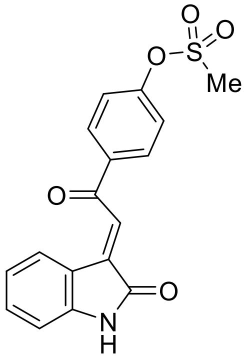	IC_{50} = 3.799 μM	Vero E6
76	**G2**	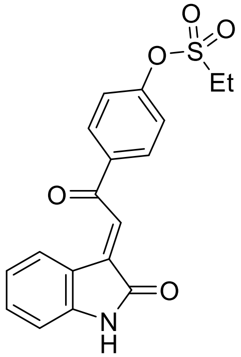	IC_{50} = 55.14 μM	Vero E6
77	**G3**	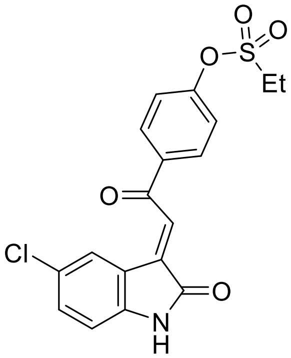	IC_{50} = 13.52 μM	Vero E6

78	**H1**	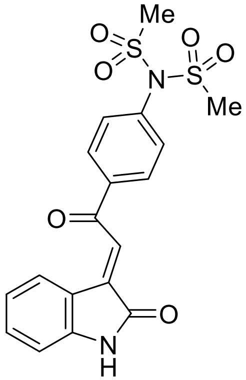	IC_{50} = 219 μM	Vero E6
79	**H2**	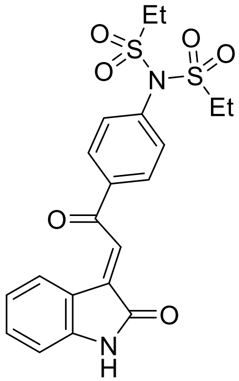	IC_{50} = 3.417 μM	Vero E6
80	**H3**	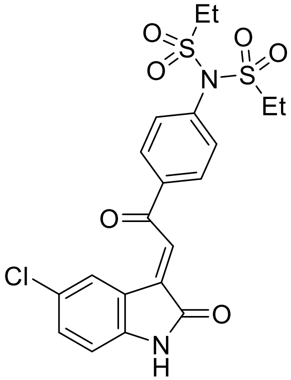	IC_{50} = 20.1 μM	Vero E6

81	**I1**	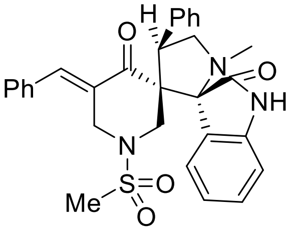	IC_{50} = 34.26 μM	Vero E6
82	**I2**	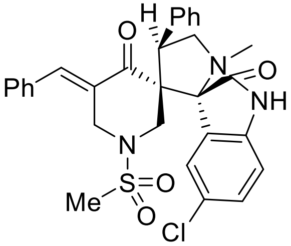	IC_{50} = 9.628 μM	Vero E6
83	**I3**	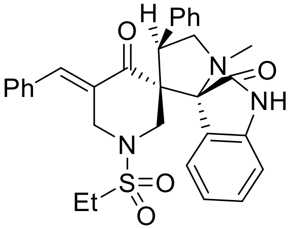	IC_{50} = 102.6 μM	Vero E6
84	**I4**	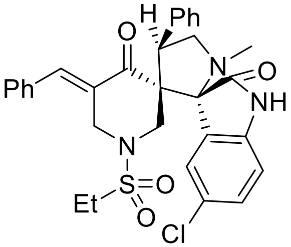	IC_{50} = 171.3 μM	Vero E6

85	**I5**	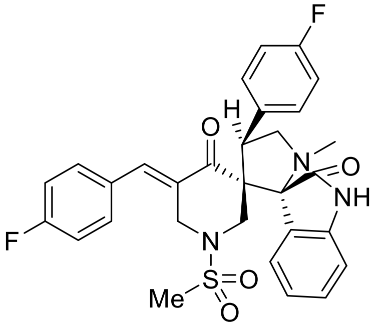	IC_{50} = 27.85 μM	Vero E6
86	**I6**	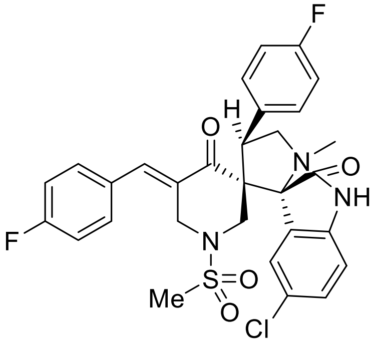	IC_{50} = 7.666 μM	Vero E6
87	**I7**	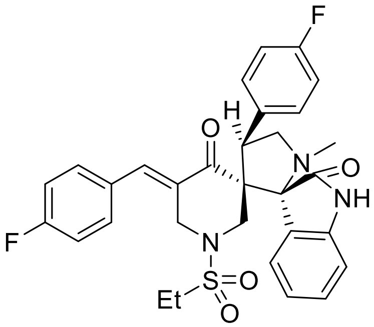	IC_{50} = 16.91 μM	Vero E6

88	**I8**	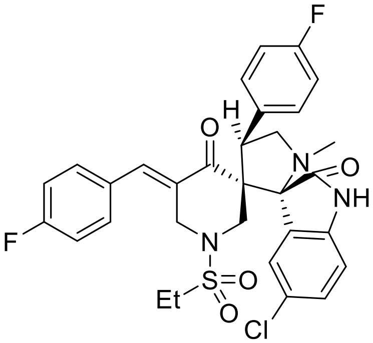	IC_{50} = 7.689 μM	Vero E6
89	**I9**	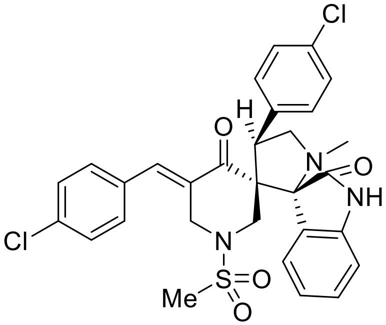	IC_{50} = 113.3μM	Vero E6
90	**I10**	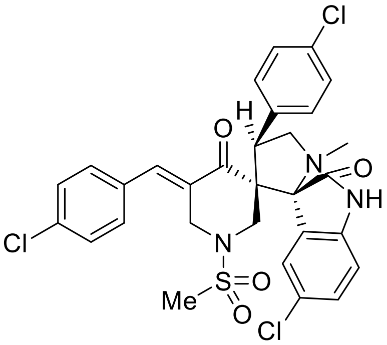	IC_{50} = 27.09 μM	Vero E6

91	**I11**	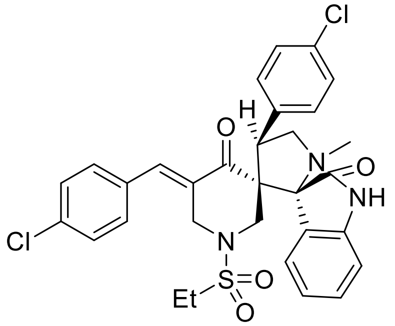	IC_{50} = 55.45 μM	Vero E6
92	**I12**	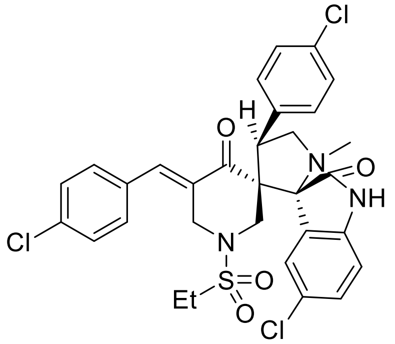	IC_{50} = 31.45 μM	Vero E6
93	**I13**	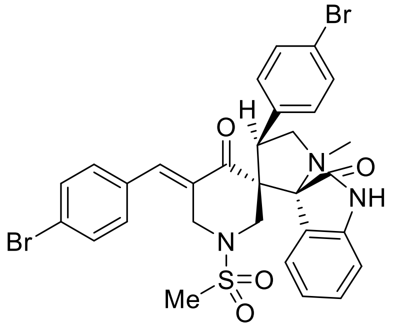	IC_{50} = 8.924 μM	Vero E6

94	**I14**	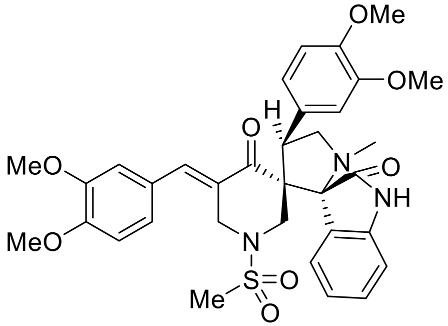	IC_{50} = 35.89 μM	Vero E6
95	**I15**	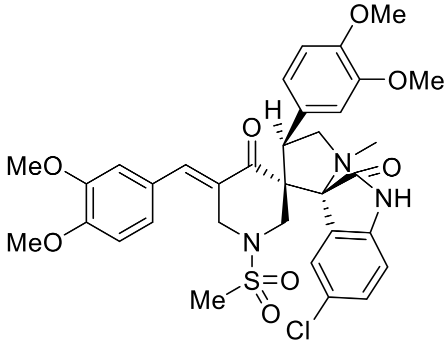	IC_{50} = 88.25 μM	Vero E6
96	**J1**	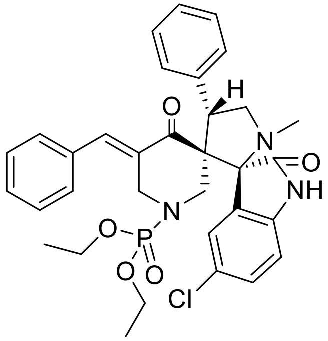	IC_{50} = 10.39 μM<br>IC_{50} = 9.605 ± 0.66 μM	SARS-CoV-2<br>M^{pro}-SARS-CoV-2

97	**J2**	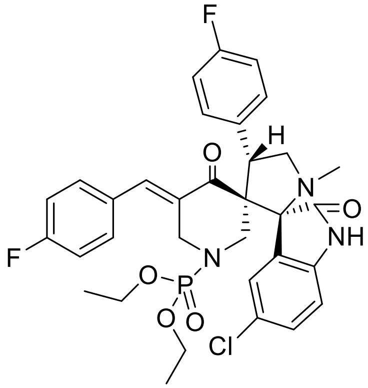	IC_{50} = 13.53 µM<br>IC_{50} = 42.82 ± 2.53 µM	SARS-CoV-2<br>M^{pro}-SARS-CoV-2
98	**J3**	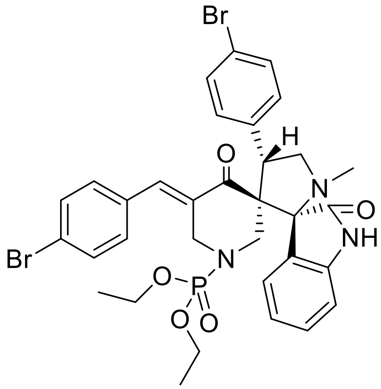	IC_{50} = 8.88 µM<br>IC_{50} = 15.59 ± 1.02 µM	SARS-CoV-2<br>M^{pro}-SARS-CoV-2
99	**22**	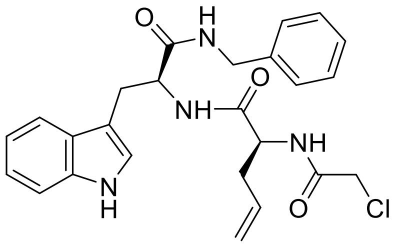	IC50 = 1.72 ±0.75 µM<br>IC50 = 0.67 ±0.59 µM	M^{pro}-SARS-CoV-2<br>PL^{pro}-SARS-CoV-2
